# A Scoping Review on Hepatoprotective Mechanism of Herbal Preparations through Gut Microbiota Modulation

**DOI:** 10.3390/cimb46100682

**Published:** 2024-10-16

**Authors:** Chin Long Poo, Mei Siu Lau, Nur Liana Md Nasir, Nik Aina Syazana Nik Zainuddin, Mohd Rahimi Ashraf Abd Rahman, Siti Khadijah Mustapha Kamal, Norizah Awang, Hussin Muhammad

**Affiliations:** Herbal Medicine Research Centre, Institute for Medical Research, National Institutes of Health, Ministry of Health Malaysia, Shah Alam 40170, Selangor, Malaysia; chinlong@moh.gov.my (C.L.P.); laums@moh.gov.my (M.S.L.); nurliana.md@moh.gov.my (N.L.M.N.); nikaina@moh.gov.my (N.A.S.N.Z.); rahimi.ashraf@moh.gov.my (M.R.A.A.R.); sitikhadijah.mk@moh.gov.my (S.K.M.K.); norizah@moh.gov.my (N.A.)

**Keywords:** hepatoprotection, herbal plants, gut microbiota, non-alcoholic fatty liver disease, steatohepatitis, scoping review

## Abstract

Liver diseases cause millions of deaths globally. Current treatments are often limited in effectiveness and availability, driving the search for alternatives. Herbal preparations offer potential hepatoprotective properties. Disrupted gut microbiota is linked to liver disorders. This scoping review aims to explore the effects of herbal preparations on hepatoprotective mechanisms, particularly in the context of non-alcoholic fatty liver disease (NAFLD), non-alcoholic steatohepatitis (NASH), and hepatic steatosis, with a focus on gut microbiota modulation. A systematic search was performed using predetermined keywords in four electronic databases (PubMed, Scopus, EMBASE, and Web of Science). A total of 55 studies were included for descriptive analysis, covering study characteristics such as disease model, dietary model, animal model, intervention details, comparators, and study outcomes. The findings of this review suggest that the hepatoprotective effects of herbal preparations are closely related to their interactions with the gut microbiota. The hepatoprotective mechanisms of herbal preparations are shown through their effects on the gut microbiota composition, intestinal barrier, and microbial metabolites, which resulted in decreased serum levels of liver enzymes and lipids, improved liver pathology, inhibition of hepatic fatty acid accumulation, suppression of inflammation and oxidative stress, reduced insulin resistance, and altered bile acid metabolism.

## 1. Introduction

The liver is the largest internal organ in the body and plays a vital role in physiological processes, making it susceptible to a wide range of toxic, microbial, metabolic, circulatory, and neoplastic insults [1]. Liver disease accounts for approximately 2 million deaths per year worldwide, which includes non-alcoholic fatty liver disease (NAFLD), hepatic steatosis, cirrhosis, viral hepatitis, and liver cancer [2], and is considered a serious health problem that significantly contributes to mortality and reduced quality of life. At present, available treatments such as colchicine, interferon, corticosteroids, and penicillamine are often limited in efficacy, are costly, and might cause adverse effects [3]. The search for alternative approaches to treat liver diseases using natural resources has become a global direction, particularly through investigating the hepatoprotective properties of herbal plants and their mechanism of actions [1].

In mammals, the gut microbiota consists of bacteria, viruses, and archaea and extends to unicellular eukaryotes like fungi and yeast [4]. The gut microbiome has a vital role in maintaining health by performing several functions, such as food fermentation, human energy metabolism, protection against pathogens, stimulating immune response, and vitamin production. It further serves to strengthen the biochemical barriers of the gut and intestine. Hence, microbiota density and composition changes can affect these functions [5]. The disruption of gut microbial homeostasis is associated with various diseases such as obesity, malnutrition, inflammatory bowel diseases, neurological disorders, cancer, liver diseases, and others [6]. Studies have shown that the disturbance of gut microbiota is related to the development and progression of liver disease. Hence, the gut microbiota could be the potential target in searching for the prevention and treatment of liver disease [7]. Interventions targeting gut microbiota for liver disease treatments include diet, prebiotics, probiotics, fecal microbiota transplantation (FMT), antibiotics, and symbiotics. However, there are no studies in the literature that have compared the direct outcomes and adverse events of prebiotics, probiotics, symbiotics, and FMT [8,9].

The use of herbal plants as an alternative source for disease treatment has been applied for many years. The presence of bioactive phytochemicals plays a major role in their therapeutic efficacy. The most commonly reported hepatoprotective mechanisms of herbal medicines include improving the cellular antioxidant defense system, exerting anti-inflammatory effects, and providing additional protection against hepatic cell injury [10]. Recent studies have demonstrated the interactions between the gut microbiota and herbal medicines in contributing to liver protection in animal models [11,12]. This review aims to systematically identify the effects of various herbal preparations on hepatoprotective mechanisms in vivo, focusing on NAFLD, non-alcoholic steatohepatitis (NASH), and hepatic steatosis involving gut microbiota modulation.

## 2. Materials and Methods

This review was conducted according to the York Framework of scoping studies by Arksey and O’Malley [13], advanced by Levac et al. [14]. This framework serves as a guide for a standardized and systematic approach to conducting scoping studies to address new or broad research questions of a complex or heterogeneous nature. As the hepatoprotective mechanisms of herbal preparations involving gut microbiota encompass heterogeneous evidence, this framework is suited to be applied. All five stages of the scoping review, namely (1) identification of research questions, (2) identification of relevant studies, (3) selection of studies, (4) data charting, and (5) collation, summarization, and reporting of findings, were undertaken. This scoping review has been registered with the Malaysian National Medical Research Register under the research ID NMRR ID-23-03336-JJR.

### 2.1. Research Questions

This review was conducted based on the primary research question, “What are the diverse effects of different herbal preparations on hepatoprotective mechanisms in vivo?” This primary question was further expanded to secondary research questions, including the following:

(i) How do these herbal preparations specifically impact liver conditions such as NAFLD, NASH, cirrhosis, and hepatic steatosis?
(ii) What role does gut microbiota modulation play in the hepatoprotective effects of these herbal preparations?
(iii) What are the challenges and limitations of the methodologies used in the in vivo animal studies?

A Population, Intervention, Comparison, and Outcome (PICO) framework was applied to address the study’s research questions (Table 1). Three main population categories were targeted to answer the three secondary research questions.

### 2.2. Search Strategy

Two reviewers independently and systematically searched studies from electronic databases, namely PubMed, Scopus, EMBASE, and Web of Science, for inclusion in this review. The search terms used were a combination of keywords such as “herbal medicines”, “medicinal plants”, “medicinal herb”, “herbal product”, “herbal plant”, “herbal extract”, “plant extract”, “phytotherapy”, “botanical drug”, “nutraceutical”, “phytopharmaceutical”, “natural product”, “phytoconstituent”, “phytochemical”, “hepatoprotection”, “liver protection”, “liver disease”, “cirrhosis”, “liver damage”, “liver injury”, “hepatotoxicity”, “gut microbiota”, “gut microbiome”, “gut bacteria”, “gut flora”, “gut axis”, “gastrointestinal microbiota” and “gastrointestinal microbiome”. The above keywords were used, catered, and adapted for each search engine. Examples of the keyword searches used for PubMed are presented in Supplementary Materials Table S1. The search strategy used a combination of free-text keywords and medical subject headings (MeSH) terms. Additional references for possible inclusion were obtained by manually searching the reference lists of eligible articles. We included all relevant studies from inception to 2023.

### 2.3. Article Inclusion

Studies were selected based on the inclusion and exclusion criteria shown in Table 2. The selection of articles was performed in two stages. In the first stage, two independent investigators screened the relevant titles and abstracts for inclusion. Titles or abstracts were screened based on inclusion and exclusion criteria. Those irrelevant articles that did not match the inclusion criteria were excluded. Any disagreement was referred to a third investigator for discussion. All relevant studies were exported into a spreadsheet, and duplicates were removed. Another two investigators retrieved full text from the titles and abstract list. In the second stage, full articles were screened to identify studies for inclusion. Another two investigators independently identified the full text for inclusion in this review. Any discrepancy was resolved through discussion with the third investigator. Data from both investigators were also compared to ensure the consistency of the review, and any discrepancies between the reviewers were discussed. Articles were excluded with reasons if they were irrelevant. Relevant articles were then assessed to answer the review questions. The results from the search were managed using Endnote X20 software, and data extracted from the full articles were documented in a Microsoft Excel spreadsheet 2016 MSO (Version 2407 Build 16.0.17830.20166).

### 2.4. Data Charting

Data extraction was carried out by two independent investigators (C.L.P. and M.S.L.), and any disparities were reviewed by a third investigator (H.M.). Two different data extraction tables were designed for in vivo articles to capture the required data for different studies and outcomes comprehensively. All investigators were briefed and trained on using the data extraction tables beforehand to ensure accurate and consistent data extraction. In general, the categories of main data extracted include the following:

(i) Article identifier: designated number, title, and author
(ii) Article characteristics: year; country; types of animal disease model; and objectives
(iii) Study population: number of animals (control groups, intervention groups); and details of study population (species and strain, age, sex, weight, and total numbers)
(iv) Intervention: plant species, plant part used, extract; formulation, route of administration, dose; duration and co-intervention
(v) Comparator: negative control, intervention description, formulation; dose and duration
(vi) Outcomes: hepatoprotective mechanisms (changes in liver enzyme levels, histological assessments of liver tissues, inflammation markers in the liver, lipid profile, and metabolic parameters; gut microbiota composition analysis, oxidative stress markers, antioxidant enzyme activities, insulin resistance, and other relevant biochemical or molecular markers)

### 2.5. Data Synthesis (Quality Assessment)

Data were synthesized qualitatively. All included studies are tabulated in Table 3 and Table 4. This scoping review was conducted and reported according to the Preferred Reporting Items for Systematic Reviews and Meta-Analyses Extension for Scoping Reviews (PRISMA-SCRs) checklist.

## 3. Results

### 3.1. Study Inclusion

**Figure 1:**
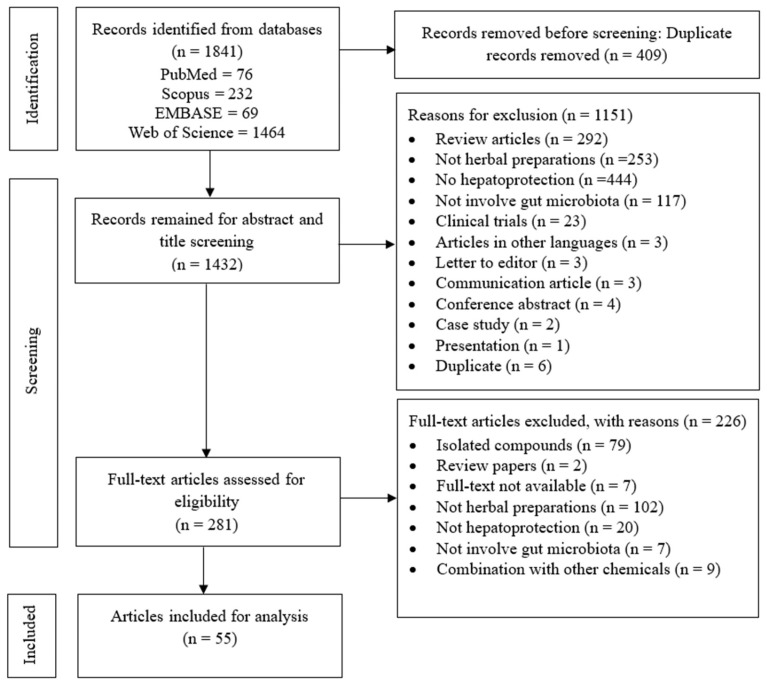
Preferred reporting items for systematic review and meta-analysis (PRISMA) flow chart of included studies.

From a total of 1841 records identified from the initial search on four selected databases, 55 articles were included in this scoping review, as presented in the preferred reporting items for systematic review and meta-analysis (PRISMA) flow chart (Figure 1). In the first stage, 409 studies were removed due to duplication. The titles and abstracts of 1432 articles were then screened for relevance, resulting in the exclusion of 1151 articles. The full texts of the remaining 281 articles were retrieved and assessed for eligibility, and ultimately, 55 studies met the inclusion criteria and were included in the scoping review. Among the 226 excluded articles, 102 studies were not herbal preparations, 79 used isolated compounds, 20 were investigating different outcomes other than hepatoprotection, 9 used herbs in combination with other chemicals, full-text was not available for 7 articles, 7 were not studying gut microbiota, and 2 were review articles.

### 3.2. Study Characteristics

Table 3 presents the characteristics of all included studies published between 2013 and 2023. All the studies were in vivo (rodent) studies. A total of 45 studies were conducted in China, with 4 studies in Japan and 1 study each in Korea, Brazil, Mexico, Canada, Israel, and Taiwan. Among the 55 articles included, most were in vivo studies reported on NAFLD (n = 46), while only 5 and 4 studies reported on NASH and hepatic steatosis, respectively. Among the 46 included studies, 38 used a high-fat diet (HFD) to induce NAFLD, 3 used a Western diet, and 4 used HFD combined with other dietary models such as cholesterol, fructose, sucrose, and glucose. There was only one study that used a mutant mouse model (ob/ob) without the use of any diet to induce NAFLD. In addition, two studies used HFD, and three studies used a methionine- and choline-deficient (MCD) diet to induce NASH, respectively. Two studies used HFD, and another two studies used a high-fat and high-sucrose diet (HFHS) and a high-fat and high-fructose diet (HFFD) to induce hepatic steatosis, respectively. In terms of the animal model, 42 studies used mice (76.4%), and 13 studies used rats (23.6%), making them the most common model organisms in the included studies. The most common mouse strains used in the 38 studies were the C57BL/6 strain (90.5%), followed by the Kunming strain (4.7%), C3H strain (2.4%), and ob/ob strain (2.4%). Among the 38 studies that used the C57BL/6 strains, one used the STAM mousee model, which was given streptozotocin. On the other hand, Sprague–Dawley rats (77%) were the most common rat strain used, followed by Wistar rats (23%). All animal models used in the studies were male.

Various herbs were used in the studies, with aqueous extracts being the most commonly used preparation, followed by enriched fractions containing specific classes of phytochemicals such as anthocyanins, polyphenols, flavonoids, and polysaccharides. Five articles reported using herbal preparations without specifying the type of extract. Generally, the dose used for herbal preparations ranged from 0.005 to 16 g/kg/day, while the dosage for isolated compounds was 10 to 800 mg/kg/day. In terms of duration, the herbs were administered for a short duration of 3 weeks and a longer duration of up to 16 weeks. All included studies were compared to the negative control groups, which were untreated HFD rats fed with mostly normal saline or distilled water. Furthermore, for assessing gut microbiota profiling, samples were collected from stool (96.4%) and colonic mucosal samples or tissue (3.6%).

### 3.3. Hepatoprotective Mechanisms

The effects and mechanisms of herbal preparations on various liver disease models by modulating gut microbiota are highlighted in Table 4. In general, herbal plants act as hepatoprotective agents by normalizing the liver biomarkers, reducing liver pathology, reducing inflammatory markers or lipid levels, modulating gut microbiota, enhancing antioxidant defense, reducing oxidative stress, mitigating insulin resistance, regulating bile acid (BA) metabolism, and other potential mechanisms. The most common mechanisms reported among all the included studies are modulation of gut microbiota (98.2%), followed by altering the liver pathology (94.5%), lipid profile (87.3%), liver enzymes (76.4%), inflammatory markers (54.5%), antioxidant and oxidative stress markers (34.6%), insulin resistance (23.6%), and BA metabolism (9.1%).

### 3.4. Disease Models

#### 3.4.1. Non-Alcoholic Fatty Liver Disease (NAFLD)

NAFLD is one of the most common causes of chronic liver diseases worldwide, particularly in Western countries. It is characterized by an irregular deposition of excessive hepatic fat in the absence of chronic viral infection and alcohol abuse [70]. The mechanisms involved in the pathogenesis of NAFLD remain enigmatic, as it is a multifactorial disease that aligns with the ‘multiple hit pathogenesis’ model. Key factors or mechanisms within this model include insulin resistance (IR), lipotoxicity, endoplasmic reticulum (ER) stress, mitochondrial dysfunction, hepatocellular apoptosis, oxidative stress, perturbation of autophagy, gut microbiota alteration, dysregulation of microRNAs, and pro-inflammatory changes [71]. Existing evidence suggests the involvement of gut microbiota in the pathogenesis and progression of NAFLD, primarily through the imbalance of gut microbiota, known as dysbiosis. This could lead to disruption in the gut–liver axis that contributes to the development of NAFLD.

Studies have shown the protective effects of herbal preparations against NAFLD through the modulation of gut microbiota (Table 4). For instance, many of the studied herbal preparations effectively prevented NAFLD in mice by decreasing ALT and AST levels, improving liver pathology, attenuating inflammation, decreasing lipid markers, modulating the gut flora, enhancing the antioxidant defense system, suppressing oxidative stress, and mitigating insulin resistance. Regarding histopathological changes in the liver, most herbs reduced hepatic steatosis and lipid accumulation in mice. For instance, *Luffa cylindrica* (*L. cylindrica*) reduced hepatocyte lipid accumulation and ameliorated hepatic steatosis in HFD-fed mice. In addition, immunohistochemical staining showed reduced monocyte chemoattractant protein 1 (MCP-1) expression levels in the liver after *L. cylindrica* administration. The results showed that *L. cylindrica* could distinctly alleviate hepatic steatosis and hepatocyte inflammation [22]. In another study, treatment with Er-Chen decoction protected against HFD-induced NAFLD in rats by reducing the serum level of pro-inflammatory factors IL-6 and IL-1β and downregulating the mRNA expression of TNF-α in the liver tissue [58]. Additionally, *Ficus hirta* Vahl. (*F. hirta*) was found to decrease the serum levels of IL-6, IL-1β, and TNF-α and suppress the upregulated proteins of TNF-α, IL-6, and IL-1β in the liver tissues of mice fed with HFD [46]. These results indicated that attenuating inflammation is one of the hepatoprotective mechanisms of herbs.

Out of the 46 studies that reported on NAFLD, 41 suggested hepatoprotective mechanisms through decreasing lipid markers. For example, defatted walnut powder extract (DWPE) from *Juglans regia* L. (*J. regia*) protected the liver from lipid injury by decreasing the liver levels of triglycerides (TGs), total cholesterol (TC), and low-density lipoprotein (LDL) and also increasing the level of HDL in HFD-fed mice [56]. Apart from altering the lipid levels, *Tetrastigma hemsleyanum* (*T. hemsleyanum*) leaves decreased the relative expression of PPAR-gamma, ACC-1, and SREBP-1c in HFD-fed mice, which are involved in lipid metabolism. Furthermore, 17 studies have been reported to alleviate NAFLD by enhancing antioxidant defense systems and reducing oxidative stress. This mechanism was shown in a study investigating the hepatoprotective effect of *Polygonatum cyrtonema* Hua polysaccharides (PCPs). The results of the study showed that PCPs improved oxidative stress by increasing the GSH and SOD activities and decreasing the MDA level in the liver of HFD-fed mice [45]. A similar finding was found in *T. hemsleyanum*, which activated the Keap1/Nrf2 signaling pathways and upregulated the genes related to antioxidant enzymes, namely NQO1 and HO-1 [23].

There were 13 studies targeting insulin resistance to protect against NAFLD. Si Miao Formula (SMF) and *Morinda citrifolia* L. (*M. citrifolia*) fruit phenolic extract have shown protective effects on NAFLD by decreasing the fasting blood glucose (FBG) and the AUC values of the intraperitoneal glucose tolerance test (IPGTT) and the intraperitoneal insulin tolerance test (IPITT) in HFHS-fed mice. The results showed that SMF and *M. citrifolia* could improve glucose tolerance and insulin sensitivity [29,36]. Moreover, four studies reported that certain herbal preparations demonstrated beneficial effects on NAFLD by regulating BA metabolism. For example, *T. hemsleyanum* extracts decreased the serum total bile acid (TBA) level in HFD-treated mice [23].

All herbs tested in the 46 included studies demonstrated hepatoprotective effects by modulating the gut microbiota. The extract of *Penthorum chinense* Pursh. (*P. chinense*), for instance, was observed to modulate intestinal microbiota by decreasing the ratio of Firmicutes and Bacteriodetes and increasing the relative abundance of *Akkermansia*, *Parabacteroides*, and *Prevotella* [15]. Additionally, Jian-Gan-Xiao-Zhi decoction (JGXZ) has been reported to improve intestinal permeability, which was attributed to the increased expressions of zonula occludens-1 (ZO-1) and occludin in the colon [39]. The hepatoprotective effects against NAFLD were also observed in mice fed with PCPs and *F. hirta*, presenting restored intestinal barrier dysfunction as a result of increased levels of short-chain fatty acids (SCFAs) [45,46].

#### 3.4.2. Non-Alcoholic Steatohepatitis (NASH)

NASH is a serious subtype of NAFLD, which is the second leading cause of liver transplant in the United States. It is characterized by hepatic inflammation and fibrosis that may progress to cirrhosis, hepatocellular carcinoma, and hepatic failure [72,73]. The pathogenesis of NASH results from a complex interplay between host and environmental factors, including the gut microbiome. Recent studies have shown that disruptions in intestinal microbiota may contribute to the pathogenesis of NASH. Altered gut permeability leads to bacterial translocation and upregulation of pro-inflammatory cytokines, ultimately leading to intestinal dysbiosis. The imbalance in the gut microbiome is believed to be the mechanism that causes the progression of NAFLD to NASH [74].

Out of 55 included studies, 5 reported the mechanisms of hepatoprotection on NASH. For example, the decreased levels of AST and ALT in the serum and liver of rats with NASH were shown by the treatment of *Tylophora yunnanensis* Schltr (*T. yunnanensis*) [61]. Furthermore, an in vivo study of pure total flavonoids from citrus (PTFC) was carried out in mice with NASH, and the results showed that PTFC reduced the degree of fat accumulation, infiltration of inflammatory cells, and ballooning of hepatocytes in the liver [62]. In another study, the Qingrequzhuo capsule (QRQZ) was administered to an MCD-induced NASH mouse model. The results showed reduced mRNA levels of pro-inflammatory cytokines (IL-1β, IL-6, and TNF-α) in the liver of the NASH mice, indicating the therapeutic potential of QRQZ on NASH [64]. Additionally, Qiang-Gan extract (QGE) was reported to decrease liver TG content and liver cholesterol in MCD-induced NASH mice [63]. Moreover, PTFC could attenuate NASH by increasing the relative abundances of Bacteroidaceae and Christensenellaceae and decreasing the relative abundance of Porphyromonadaceae and Streptococcaceae [62]. In addition, QRQZ reduced colonic permeability by increasing the expression of ZO-1 and occludin in the colon. In terms of oxidative stress indicators, the SOD and GSH-Px activities increased, and the MDA levels decreased after QRQZ treatment [64]. Furthermore, the liver and serum BA concentrations were decreased in the QGE-treated mice [63]. The same finding was observed in another study using the Salvia-Nelumbinis naturalis (SNN) formula. The results of that study reported that SNN decreased the total fecal BA level and increased the colonic nuclear BA receptor and FXR expression, suggesting that modulating BA concentration might be a novel strategy to protect against NASH [65].

#### 3.4.3. Hepatic Steatosis

Hepatic steatosis is defined as intrahepatic fat of at least 5% of liver weight, and it can be characterized by the increased accumulation of lipids in the liver. The prolonged accumulation of triacylglycerol may lead to hepatic inflammation, liver metabolic dysfunction, and advanced forms of NAFLD. Several mechanisms are involved in hepatic steatosis, including increased lipolysis from the fat cells, increased flux of fatty acids to the liver, increased de novo lipogenesis, and/or reduced clearance through *β*-oxidation or very low-density lipoprotein secretion [75]. The development of hepatic steatosis has also been associated with dysbiosis [76].

Various herbs have shown beneficial effects on hepatic steatosis via gut flora alteration (Table 4). For instance, aguamiel concentrate (AC) from *Agave salmiana* (*A. salmiana*) was shown to reduce hepatic steatosis by increasing plasma ALT, decreasing hepatic lipid accumulation, reducing hepatic TNF-α, and decreasing MDA [66]. Arctic berry extracts (ABEs) were found to ameliorate steatosis in mice by alleviating hepatic inflammation, as evidenced by decreased mRNA expression of Tlr4. Additionally, ABEs were reported to reduce HFHS-induced hepatic triacylglycerol accumulation and triacylglycerolemia. In terms of gut microbiota composition, ABEs decreased the Firmicutes/Bacteroidetes (F/B) ratio, increased the abundance of Peptostreptococcaceae, *Akkermansia muciniphila*, and *Turicibacter*, and lowered the presence of *Lactobacillus* and *Bifidobacterium* in the fecal microbiota. In addition, an increased tyrosine-phosphorylated CEACAM-1 level was observed in ABE treatment. This result suggests that ABEs protect the liver from the detrimental effects of HFHS feeding, thus preventing hyperinsulinemia from diet-induced obesity (DIO) [68]. Furthermore, apple polyphenol extract (APE) improved hepatic steatosis induced by a high-fat diet in C57BL/6 mice. This effect was achieved by reducing the total fecal BA content, particularly primary BA levels, such as cholic acid, chenodeoxycholic acid, and muricholic acid [67].

## 4. Discussion

### 4.1. Study Characteristics

This is the first review on herbal preparations, consisting of 55 included studies published from 2013 to 2023, summarizing existing in vivo research on their hepatoprotective effects via modulation of gut microbiota. Notably, NAFLD, followed by NASH and hepatic steatosis, emerged as the primary disease models studied, likely due to NAFLD’s high prevalence [77]. NAFLD progresses to NASH in 20–30% of cases and may further advance to cirrhosis and hepatocellular carcinoma (HCC). Previous studies have shown that intestinal dysbiosis has been linked to NAFLD progression to NASH [78], with hepatic steatosis serving as an early stage of NAFLD [79].

NAFLD’s multifactorial etiologies, often stemming from unhealthy lifestyles like hypercaloric diets, are frequently associated with high fat and fructose intakes [80]. There are many dietary models available to induce liver diseases, such as high-fat diets, high-fructose or -sucrose diets, combined diets, and nutrient-depleted diets (e.g., MCD diet) [81]. The current review revealed that HFD with fat content ranging from 30% to 60% is prevalent in inducing NAFLD, NASH, and hepatic steatosis [82]. A study by Xu et al. [83] has shown that an HFD consisting of 30% fat successfully induced NASH, as indicated by the increased levels of liver weight and liver index. Similarly, our results indicated a remarkable increase in liver index and lipid accumulation in HFD-induced NAFLD mice and rat model groups [41,58].

Apart from HFD, high-fructose or -sucrose and MCD diets are used to induce liver diseases in this review. Excessive fructose or sucrose consumption drives de novo lipogenesis and is linked to NAFLD, obesity, and insulin resistance [84]. Yustisia et al. [85] demonstrated that a high-fat and high-fructose diet induces hepatic steatosis, dyslipidemia, renal lesions, and hyperuricemia in non-obese rats, akin to our study demonstrating hepatic steatosis in Kunming mice fed with a high-fructose diet [69]. MCD diets comprise high sucrose (40%) and fat (10%) but lack methionine and choline, which are essential for hepatic mitochondrial β-oxidation and very low-density lipoprotein (VLDL) synthesis [86]. When animals are fed an MCD diet, the rapid accumulation of TGs in the liver occurs, leading to NASH followed by fibrosis [87]. The study by De Lima et al. [88] used an MCD diet and reported the development of histological NASH with cirrhosis and CK-19-positive hepatocellular carcinoma. Moreover, Kirsch et al. [89] demonstrated that C57/BL6 male mice exhibited histological characteristics closely resembling those observed in humans with NASH after feeding with an MCD diet. Our review similarly found NASH modeled in C57BL/6 mice via MCD diets [63].

This scoping review revealed that the majority of the studies used aqueous extract against liver diseases, especially NAFLD, as it is environmentally friendly and easily accessible [90]. Water is typically used to extract polar phytochemicals such as phenolics, alkaloids, and flavonoids, generally known for hepatoprotective effects [91]. Nevertheless, the extraction methods did not interfere with the hepatoprotective effect of the herbal preparations. For instance, both of the ethanolic and aqueous extracts of *Terminalia belerica* significantly prevented the physical and biochemical changes in ethanol-induced hepatotoxicity in rats [23,60,92]. Furthermore, in order to study the therapeutic effects of herbal preparations, the duration of the treatment plays an important role in hepatoprotective potential. The selected studies utilized different treatment durations, and a short treatment duration of 3 weeks was able to demonstrate hepatoprotective effects [17]. The different treatment durations are believed to be related to the concentration of the herbal preparations, with varying doses observed across the selected studies.

Recent studies emphasize the close relationship between the gut microbiota and health. Microbiota distribution varies based on health, diet, and lifestyle, with changes reflecting disease states [11]. For 16s RNA sequencing analysis, various types of samples, including stool, mucosal swabs, and colonic swabs, are used to determine gut microbiota profiling. The current review revealed that stool samples were predominantly used for gut microbiota profiling due to their non-invasive collection and convenience for repeated sampling [93]. In contrast, only two studies opted for colonic mucosal samples, primarily due to the costliness and invasiveness of this sampling method, which might cause unexpected bleeding and infection [94].

### 4.2. Regulation of Gut Microbiota

Based on current studies, herbal preparations can regulate the composition of gut microbiota and the levels of gut microbial metabolites to improve diseases. In this review, some herbal preparations have been shown to reduce the F/B ratio. Firmicutes and Bacteriodetes are the most abundant bacteria at the phylum level. An elevated ratio of F/B in the gut microbiota has been associated with metabolic diseases such as obesity, hyperlipidemia, and NAFLD [95]. Therefore, decreasing the F/B ratio would improve the disease conditions. Moreover, *Bacteroidetes*, *Lactobacillus*, *Bacillus*, *Akkermansia*, *Parabacteroides*, and *Prevotella* are shown to be beneficial to the host and referred to as beneficial gut microbiota (BGM) [11]. *Akkermansia*, for instance, protects the intestinal mucosal barrier by degrading mucin and producing propionic acid [96]. *Lactobacillus* bacteria possess probiotic properties that help regulate host health and are used to treat dyslipidemia [97]. *Parabacteroides* and *Prevotella* are anti-inflammatory symbionts that affect immune system metabolic pathways [98]. Our results demonstrated that *Cannabis sativa* L. (*C. sativa*), DCH decoction, *M. citrifolia*, *Myristica fragrans* Houtt (*M. fragrans*), *P. chinense*, JGXZ decoction, and other herbs significantly increased BGM abundance in HFD-induced NAFLD mice [15,31,33,36,38,39].

In addition to increasing the relative abundance of BGM, herbal preparations decreased harmful gut microbiota (HGM) such as *Lachnoclostridium*, Enterobacteriaceae, *Bacillus*, *Shigella*, *Helicobacter*, and other microbiomes in our findings [11]. *Lachnoclostridium* is a mucin-degrading bacteria that can impact both glycan composition and mucus thickness, and its increased abundance is associated with NAFLD in rat models [99]. Enterobacteriaceae has been recently demonstrated to be associated with the progression of NAFLD [100]. A previous study demonstrated that the abundance of *Bacillus*/*Shigella* was significantly increased in HFD rats but decreased in the Qushi Huayu Fang-treated rats [44]. Moreover, sulforaphane, a major chemical constituent of *Raphanus sativus,* was reported to inhibit the growth of HGM such as *Bacillus coli*, *Shigella*, and *Helicobacter pylori*, without affecting the abundance of *Lactobacillus* levels [101]. All these findings indicate that decreasing the abundance of HGM could be the ameliorative mechanism of herbs on NAFLD.

A disrupted intestinal barrier can lead to the pathogenesis of NAFLD. When the intestinal barrier is damaged, endotoxins such as lipopolysaccharide (LPS) from gut microbes could escape from the intestinal lumen into the bloodstream via the hepatic portal system [34]. LPS, a crucial component of the outer membrane of Gram-negative bacteria, increases gut permeability, binds to CD14 and Toll-like receptor 4 on the surface of immune cells, activates the nuclear factor kappa B (NF-κB) signaling pathway, and then triggers a series of inflammatory processes that ultimately lead to chronic low-grade inflammation. Thus, improving the function of the intestinal barrier and suppressing inflammation are the mechanisms to ameliorate NAFLD [102]. Certain herbal preparations have been shown to restore the intestinal barrier function by inhibiting LPS production, primarily by targeting LPS-producing bacteria such as *Desulfovibrio*, *Escherichia–Shigella*, *Enterococcus*, *Klebsiella*, and *Enterobacter* [95]. In our findings, *M. citrifolia* fruit phenolic extract caused a significant reduction in the abundance of *Helicobacter*, Desulfovibrionaceae, *Desulfovibrio*, and *Bacteroides vulgatus* species in HFD-fed mice [36]. Desulfovibrionaceae, a family of sulfate-reducing bacteria (SRB), and Desulfovibrio, a genus of SRB, are reported to be a type of LPS-producing bacteria that are capable of inducing an inflammatory response and damaging intestinal epithelial cells. It has been reported that *Desulfovibrio* is associated with NAFLD, obesity, and inflammation [34]. Our results were in corroboration with previous research conducted by others who discovered that *Dendrobium officinale* improved liver recovery in NAFLD mice by reducing the relative abundance of *Desulfovibrio* by 76.1% and the level of microbial LPS [103]. Additionally, the number of Desulfovibrionaceae bacteria was significantly reduced by *Momordica charantia* L. (*M. charantia*) in obese rats [104]. In the present research, the abundance of *Escherichia*/*Shigella* was reduced in HFD-induced NAFLD rats after DZD treatment [34]. *Escherichia*/*Shigella* is another group of Gram-negative bacteria that contain LPS in their cell walls and contribute to the high serum LPS and TNF-α expression usually found in NAFLD patients. An increase in *Escherichia*/*Shigella* can trigger intestinal inflammation, causing intestinal barrier dysfunction [44].

The interaction between gut microbiota and intestinal epithelial cells plays a crucial role in gastrointestinal tract homeostasis, modulated by tight junctions [105]. Tight junctions are the dynamic permeability barrier of intestinal epithelial cells that prevent the transmission of harmful substances from causing liver damage and inflammation. Decreased expression of tight junction proteins, such as ZO-1, occludin, and claudin-1, can impair intestinal barrier function and increase gut permeability, contributing to the inflammatory response [95]. ZO-1 is a cytoplasmic plaque protein that can connect the transmembrane proteins to the cytoskeleton, while occludin can influence the tight junction of intestinal epithelial cells by regulating macromolecule flux [106,107]. In this study, JGXZ protected the intestinal mucosal barrier by upregulating the expressions of ZO-1 and occludin [39]. These results were in accordance with a study that used polyphenol-rich loquat fruit extract (LFP), which improved HF-induced breakage of the intestinal barrier by increasing the protein expression of ZO-1 and occludin [18]. In addition, increased levels of ZO-1 and occludin were observed in HFD rats after WGHP, Simiao decoction, and Quzhuo Tongbi decoction treatments, indicating that herbs could prevent the accumulation of endotoxin in blood by protecting the intestinal physical barrier [19,29,108].

Gut microbiota produces bioactive metabolites such as SCFAs and BAs. SCFAs are gut microbiota-derived metabolites produced from the fermentation of polysaccharides. SCFAs, including acetic acid, propionic acid, and butyric acid, are beneficial in improving metabolic diseases [95]. SCFAs alleviate gut inflammation and improve intestinal permeability. Studies have shown that SCFAs, specifically butyrate, can reduce the production of IL-6, MCP-1, and TNF-α and inhibit the NF-κB signaling pathway [109] Furthermore, butyric acid and propionic acid can improve glucose homeostasis by activating intestinal gluconeogenesis through the cAMP-dependent pathway and thus reducing hepatic glucose production [110]. Acetic acid improves lipid metabolism by inhibiting liver lipid accumulation through the upregulation of the peroxisome proliferator-activated receptor α (PPARα) gene and several fatty acid oxidation-related proteins [111]. In addition, SCFAs promote insulin secretion and sensitivity while also reducing energy intake and insulin resistance [112,113].

In a recent study, PCPs and *F. hirta* and *T. hemsleyanum* leaf extracts improved intestinal barrier function by targeting the gut microbiota and thus increasing SCFA levels [23,45,46]. All these herbs increased the relative abundance of *Ruminococcaceae*, *Bifidobacteriales*, *Allobaculum*, *Akkermansia*, *Faecalibaculum*, *and Butyricicoccus*, which have been identified as SCFA-producing bacteria [114,115]. This is consistent with our findings that showed DZD treatment decreased the abundance of SCFA-producing bacteria (*Ruminococcaceae*, *Oscillibacter*, and *Butyricicoccus*) in NAFLD rats [34]. These SCFA-producing bacteria prevent metabolic endotoxemia by strengthening the intestinal barrier. Changes in these gut bacteria induce an increment of SCFAs, decreasing intestinal permeability and contributing to intestinal homeostasis [116]. In addition, Han et al. [29] demonstrated that the Simiao decoction could increase the abundance of SCFA-producing bacteria (*Bifidobacterium* and *Faecalibaculum*) and improve lipid metabolism and inflammation in NAFLD rats. Moreover, *Roseburia*, which is another SCFA-producing bacterium, has been proven to be associated with type 2 diabetes mellitus (T2DM), obesity, and inflammation [117].

### 4.3. Liver Biomarkers

Hepatoprotection is often reflected in the normalization of liver biomarkers and a reduction in liver pathology. Biomarkers are measurable indicators that provide information about the physiological state of an organ. Many herbal interventions aim to restore the levels of liver biomarkers to their normal range. These biomarkers include enzymes such as alanine aminotransferase (ALT) and aspartate aminotransferase (AST), which, when elevated, suggest liver cell damage. Alkaline phosphatase (ALP) and gamma-glutamyl transferase (GGT) are other enzymes indicative of liver function and biliary health. Chronic liver diseases often involve inflammation, fibrosis, and, in advanced stages, cirrhosis. All these biomarkers, especially ALT and AST, were decreased in our findings. A reduction in ALT and AST is usually observed after herbal intervention, and it is the hepatoprotective mechanism of herbs to improve liver function. In addition, the gut microbiota plays an important regulatory role in hepatic function and affects the severity of liver diseases. A previous study showed that the abundance of Firmicutes was negatively correlated with ALT and AST levels. A reduction in the abundance of Firmicutes might be associated with worsened hepatic function [118]. This contradicts our findings, which show that the abundance of Firmicutes was reduced, resulting in low ALT and AST levels.

### 4.4. Liver Histopathology

Herbal interventions that lead to a reduction in liver pathology demonstrate their ability to mitigate these adverse structural changes caused by liver diseases. This can be assessed through histological examinations of liver tissue, where a decrease in inflammatory infiltrates, fibrotic tissue, and other pathological features indicates a positive response to the herbal treatment. This was observed in our findings, where the herbal preparations improved the liver histopathological changes of mice with liver diseases. This hepatoprotective mechanism is closely related to the regulation of the gut microbiota, as gastrointestinal dysbiosis is one of the factors connected to the liver pathological changes, especially fat deposition in the liver [119]. In NAFLD, the decreased abundance of Bacteroidetes and increased abundance of Firmicutes and *Proteobacteria* were reversed by the herbal preparations in our findings, which results in improved liver histopathological changes such as reduced liver lipid droplet accumulation and improved steatosis [120].

### 4.5. Lipid Metabolism

Disruptions in lipid metabolism, common among NASH patients, lead to the accumulation of various lipids like TC and TGs, alongside increased levels of ALT and AST in the liver, exacerbating liver damage and elevating the risk of cardiovascular diseases, particularly in individuals with T2DM and NAFLD [121,122]. Research indicates that Christensenellaceae levels are reduced in individuals with metabolic syndrome (MetS) compared to healthy counterparts, implying its potential significance in metabolic health and suggesting potential avenues for therapeutic exploration [123]. TGs, the primary form of fat accumulated in NAFLD, play a crucial role in the pathogenesis of the condition. Dysregulation in hepatic de novo lipogenesis, free fatty acid (FFA) availability, and lipid export contribute to hepatic fat deposition, with TG levels associated with insulin resistance, a key factor in NAFLD development. While TG accumulation alone may not be hepatotoxic, the enzyme DGAT2 exacerbates steatohepatitis by reducing TGs and enhancing FFA oxidation. The dysregulation of lipolysis and de novo lipogenesis are particularly critical in the association between insulin resistance and NAFLD. Lycium barbarum oligosaccharides (LBOs) demonstrated efficacy against hepatic steatosis by significantly reducing TGs and improving liver lipid accumulation compared to the HFD group in mouse models [124].

The gut microbiota has been associated with cardiovascular diseases (CVDs) due to their involvement in cholesterol metabolism. It can change the blood lipid composition through its role in BA metabolism and the generation of microbial products such as primary and secondary BAs, trimethylamine N-oxide (TMAO), and SCFAs [125]. A higher abundance of *Lactobacillus* and *Enterobacteriaceae*, a lower abundance of Bacteroidetes, and an increased F/B ratio correlate with CVD. Most of these gut microbiomes may increase gut permeability, leading to increased systemic levels of gut microbial metabolites, which, in turn, cause low-grade chronic inflammation. This inflammation may then alter plasma lipid and lipoprotein levels [126]. Therefore, the herbal preparations in our findings restored the microbial population by decreasing the abundance of *Lactobacillus* and *Enterobacteriaceae,* as well as the F/B ratio, causing a decrease in the serum levels of TC, TGs, and LDL-c. LBOs regulated the modulation of HFD-induced shifts in the intestinal flora, decreasing the abundance of *Barnesiellaceae*, *Barnesiella*, and CHKCI001 while increasing the proportion of *Dubosiella*, *Eubacterium*, and *Lactobacillus* [124].

### 4.6. Insulin Resistance

Insulin resistance, a central pathogenic feature of metabolic syndrome and a common risk factor for NAFLD, exists in systemic and hepatic forms. Systemic insulin resistance impedes glucose uptake, and hepatic insulin resistance disrupts insulin’s suppression of hepatic glucose production while stimulating lipogenesis. Genetic variations in insulin signaling, such as the ENPP1 Lys121Gln and IRS1 Gly972Arg polymorphisms, are associated with NAFLD pathogenesis, adding complexity to the condition. Herbal interventions targeting insulin resistance and regulating BA metabolism show promise in ameliorating NAFLD progression [127]. Moreover, the gut microbiota has been reported to contribute to insulin resistance. The changes in the gut microbiota increased the absorption of LPS, which activates the Toll-like receptor (TLR), leading to increased activation of the innate immune system and inflammatory pathways [128]. As a result, insulin signaling is impaired with decreased phosphorylation of the insulin receptor (IR), insulin receptor substrate (IRS), and Akt, as well as decreased tyrosine phosphorylation of IRS-1 [129]. Previous studies showed that the increased presence of Bacteroidetes in the gut microbiota was negatively correlated with insulin resistance. Bacteroidetes was found to be enriched in the gut of insulin-sensitive individuals, and it reduced fecal monosaccharide accumulation, resulting in lowered lipid accumulation and inflammation, which contributes to reduced insulin resistance [130]. This is in agreement with our findings that the F/B ratio was reduced and Bacteroidetes was increased, which resulted in decreased serum insulin and Homeostatic Model Assessment of Insulin Resistance (HOMA-IR) levels and enhanced insulin sensitivity after herbal intervention.

### 4.7. Bile Acid Metabolism

BAs are essential molecules in modulating lipid absorption and cholesterol homeostasis. BAs also play a key role in regulating not only their synthesis but also hepatic lipid and energy homeostasis. They are produced by the liver to form the primary BAs and converted to secondary BAs by the gut microbiota [131]. The interactions of BAs and the gut microbiota are bidirectional. Changes in gut microbiota abundance can alter BA composition, influencing the microbiota composition and leading to dysbiosis, with impaired BA signaling evident in patients with NAFLD and dysbiosis. Since BAs and gut microbiota interact reciprocally, modulating them might be a promising herbal strategy for treating liver diseases [120]. In the present study, MCD diet-induced NASH mice who received herbal treatment showed a significant decrease in serum and TBA levels, indicating that herbal treatment modulated the BA metabolism. Furthermore, bile salt hydrolase (BSH) catalyzes the conversion of primary BAs to secondary BAs. BSH-producing microbiomes such as *Bacteroidetes*, *Clostridium*, *Bifidobacterium*, *Listeria*, *Lactobacillus*, *Eubacterium*, *Escherichia*, and *Ruminococcus* have been reported to be involved in BA conversion [132]. Our findings show that the abundance of Bacteroidetes and Clostridium increased in herbal plant-treated mice, contributing to the increased lithocholic acid (LCA) production, suggesting that the underlying possible mechanisms of herbal treatment in preventing NASH may be mediated by the interaction of BAs and the gut microbiota [63].

### 4.8. Antioxidant Activity and Oxidative Stress

Beyond biomarkers and tissue-level changes, herbal interventions may exert protective effects at the cellular level by preventing oxidative damage, promoting cell survival, and enhancing the regenerative capacity of liver cells. Liver diseases often involve increased cell death through apoptosis or necrosis. Herbal treatments may influence these cell death pathways to promote a balance that supports cell survival. Herbal interventions with anti-inflammatory properties can attenuate hepatic inflammation, which is particularly relevant in conditions like NASH and viral hepatitis, where inflammation is a key driver of disease progression [127]. Studies have shown that altered gut microbiota or intestinal dysbiosis can lead to inflammation and increase oxidative stress. Dysbiosis causes increased intestinal permeability, and elevated permeability triggers the liver’s inflammatory response [131,133]. An alteration in the gut microbiome can cause a shift in the redox status of an organism, as gut bacteria are capable of inducing ROS production that leads to oxidative stress [134]. Thus, the decrease in inflammatory and oxidative stress markers shown in our findings might be the outcome of the hepatoprotective effects of herbal preparations through the regulation of gut microbiota. The reduced level of liver MDA found in rats fed an HF diet after herbal treatment suggests that herbal treatment inhibited the formation of ROS. Several studies have demonstrated that Nrf2 acts as a regulator of cellular antioxidant response. In response to oxidative stress, Nrf2 translocates to the nucleus and activates the transcription of several genes, such as GSH-Px and SOD. In our findings, herbal preparations increased the protein expression of Nrf2 and GSH-Px and SOD activities in the liver of HF-fed rats, indicating that herbal preparations prevent liver oxidative stress by modulating the Nrf2 signaling pathway [25,135]. As discussed earlier, SCFAs have been shown to inhibit oxidative stress and inflammation. Propionic acid and butyric acid can decrease the production of pro-inflammatory factors such as MCP-1 and pro-inflammatory cytokines such as TNF-α and IL-6 [109]. Our findings show that herbal preparations increased the production of SCFAs and increased the relative abundance of SCFA-producing bacteria such as *Ruminococcaceae*, *Bifidobacteriales*, *Allobaculum*, *Akkermansia*, and *Prevotellaceae* [19,23]. These data suggested that herbal preparations modulated gut microbiota and SCFA composition, resulting in reduced inflammation and oxidative stress.

### 4.9. Inflammation

A large number of cytokines have been demonstrated to play important roles in regulating liver injury, inflammation, fibrosis, and regeneration [136]. Culture broth extract of *Penicillium polonicum* (endophytic fungi of *P. nigrum* fruit) was found to inhibit pro-inflammatory cytokines by controlling the TNF-α, IL-6, IL-1β, iNOS, MPO, and NO levels [135,137]. The primary cause of liver-related mortality in NAFLD patients is the accumulation of extracellular matrix in the liver, leading to progressive fibrosis, cirrhosis, portal hypertension, and liver failure. The fibrogenesis process is initiated by signaling from stressed or injured hepatocytes and activated macrophages, specifically Kupffer cells in the liver, prompting the transformation of resident hepatic stellate cells into myofibroblasts. These myofibroblasts then produce matrix proteins at a rate faster than degradation, contributing to fibrosis [138]. There is a growing understanding of NASH-specific fibrogenic pathways [139]. In mouse models of NASH, heightened signaling by the transcriptional activator TAZ in NASH hepatocytes is observed, promoting the secretion of Indian hedgehog ligand. This secretion leads to paracrine fibrogenic signaling to stellate cells [140]. Another NASH-specific pathway involves the PNPLA3-I148M variant directly affecting stellate cell fibrogenesis [141]. This suggests that therapies targeting PNPLA3 function could have antifibrotic effects [142]. Herbal preparations downregulate pro-inflammatory cytokines (IL-1β, IL-6, and TNF-α) in the liver. They also reduce colonic permeability, enhance the expression of tight junction proteins (ZO-1 and occludin), and modulate oxidative stress indicators, as evidenced by increased SOD and GSH-Px activities and decreased MDA levels.

### 4.10. Future Perspectives on Gut Microbiota-Related Strategies

Gut microbiota-related strategies are emerging as potential approaches to treating liver diseases, given the strong connection between the gut and liver, known as the gut–liver axis. These strategies include diet, probiotics, prebiotics, synbiotics, and fecal microbiota transplantation (FMT) [143]. FMT is a procedure that restores a balanced gut microbiota by transferring feces from a healthy donor to a patient’s gastrointestinal tract (GI) [144]. FMT has been widely utilized in the treatment of several liver diseases, such as NAFLD, autoimmune hepatitis (AIH), and hepatocellular carcinoma (HCC). Zhou et al. showed that an 8-week FMT intervention could restore gut microbiota balance and reverse steatohepatitis in mice fed with an HFD, as indicated by a significant decrease in intrahepatic lipid accumulation, triglycerides, and pro-inflammatory cytokines. Additionally, body weight, liver index, fasting blood glucose (FBG), and HOMA-IR were improved, suggesting a beneficial effect of FMT on HFD-induced metabolic disturbances [145]. Subsequently, a clinical study indicated that the clinical symptoms of NAFLD in patients undergoing FMT were significantly improved compared to patients in the no-FMT group [146]. However, standards related to microbiota quality and safety control have not been widely accepted. FMT is still in the preclinical stage for liver disease patients, thereby deserving further clinical investigation. The safety of FMT, including the short-term and long-term adverse effects on liver diseases and the parameters required for post-transplant follow-up, remains unknown [144]. Increasing the availability of clinical data on FMT for liver diseases will enhance our mechanistic understanding in a field that has previously lacked substantial information.

Studies have shown the interaction of herbs and the gut microbiota in treating liver diseases. Herbs can regulate the composition of the gut microbiota and the levels of gut microbial metabolites to improve disease outcomes [95]. Hence, they can be considered part of gut microbiota-related strategies to manage and treat liver disease. Many herbs have been shown to not only regulate gut microbiota but also to possess antioxidant and anti-inflammatory properties. For instance, *Salviae miltiorrhizae* (*S. miltorrhizae*), *Brassica juncea* (*B. juncea*), *T. hemsleyanum*, and other herbs in our review were reported to modulate the gut microbiota, leading to decreased hepatic lipid accumulation, triglycerides, and pro-inflammatory cytokines and thus improving NAFLD conditions. Improvement in liver index and insulin resistance were also observed in HFD-induced NAFLD rats following herbal treatment [20,21,23]. These findings are similar to the results shown in the HFD-induced steatohepatitis mouse model after FMT intervention. This suggests that herbal preparations are as effective as FMT in modulating gut microbiota for treating liver diseases, with additional medical properties that could benefit liver health. Herbs can complement other gut microbiota-related treatments, especially FMT, by providing anti-inflammatory and antioxidant benefits for enhanced efficacy. Nevertheless, more preclinical and clinical investigations are required to confirm the safety and efficacy of herbal preparations and FMT in treating liver diseases through gut microbiota modulation.

## 5. Study Challenges and Limitations

This review has several limitations. Firstly, only English articles with full-text accessibility were included, potentially excluding valuable findings published in other languages and leading to an incomplete understanding of the subject matter. Secondly, due to insufficient in vitro studies and clinical trials meeting the inclusion criteria, in vitro and clinical studies were excluded. Although the limited number of such studies identified might overlook useful in vitro and human data concerning gut microbiota, the substantial inclusion of animal studies is believed to adequately represent the bulk of available evidence on hepatoprotective mechanisms of herbs specific to gut microbiota regulation. Thirdly, the scope of the review was limited to herbal extracts and mixtures, omitting compound-based interventions, as the therapeutic effect of herbal preparations often stems from the combined effects of multiple phytochemicals present in the whole plant. Therefore, studies on single or isolated compounds were excluded. Fourthly, articles investigating herbal preparations in combination with other chemicals, such as synthetic compounds and prebiotics, were also excluded. Lastly, the quantitative analysis of adverse reactions was not feasible in this review due to the lack of reported adverse effects data in the included studies.

Despite advances in the research of herbal medicines and their effects on the gut microbiota, the standardization of herbal preparations and the identification of the active constituents responsible for hepatoprotective effects are intrinsically complex. The interactions between individual phytochemicals and diverse gut microbiota constituents remain significant hurdles. Further analysis of more chemical composition is necessary to better understand the overall impact of herbal medicines on the gut microbiota. Humans, mice, and rats share some similarities in their gut microbiota composition, particularly at the phylum level, with Firmicutes and Bacteroidetes being dominant in all three. However, there are significant differences in the specific genera and species present, as well as in the microbiota’s overall diversity and functional capacity. These differences are influenced by diet, environment, and host genetics. As a result, the effects observed in rodent models may not fully translate to humans. Moreover, translating preclinical results into clinical efficacy poses a challenge. Humans, mice, and rats share similarities in their gut microbiota composition at the phylum level but not at the species level. These differences are influenced by diet, environment, and host genetics. As a result, the effects observed in rodent models may not fully translate to humans [147]. Clinical trials are essential to provide practical and safe strategies to treat liver disease using herbal medicines by modulating the gut microbiota. In addition, the interindividual variability in the gut microbiota and host responsiveness makes it difficult to predict how herbal interventions may influence gut microbiota composition and host outcomes. This may, in turn, present a challenge in creating generalized treatments and underscores the need for personalized approaches. The consistency of results among studies and the efficacy of a herbal intervention in eliciting beneficial changes to the gut microbiota might also be affected. Furthermore, advancing high-throughput sequencing technologies and metabolomics will provide a deeper understanding of the gut microbiota and elucidate the complex mechanisms underlying the action of herbal medicine on the gut microbiota.

## 6. Conclusions

In conclusion, the gut microbiota closely correlates with liver diseases, especially NAFLD, NASH, and hepatic steatosis. Changes in gut microbiota influence the pathogenesis and progression of liver diseases, making targeting the gut microbiota a promising treatment strategy. Herbal preparations have shown hepatoprotective effects and are important in treating liver disease. This review demonstrated that the hepatoprotective effects of herbal preparations are closely related to their interaction with the gut microbiota. Herbal preparations protect the liver from hepatic diseases by ameliorating gut dysbiosis, restoring gut barrier integrity, promoting gut-derived SCFA production, attenuating increased serum liver enzymes and lipids, improving liver pathology, inhibiting hepatic fatty acid accumulation, suppressing inflammation and insulin resistance, restoring antioxidant status, reducing oxidative stress, and altering BA metabolism. Overall, the roles of herbal preparations in the hepatoprotection of liver diseases, specifically NAFLD through the modulation of the gut microbiota, is encouraging. A comprehensive understanding of the hepatoprotective mechanisms of herbal preparations targeting intestinal microbiota will help develop therapeutics for preventing and treating liver diseases. Further studies are warranted to study the mechanisms of action of effective components in herbal preparations with hepatoprotective effects. This exploration can help identify new targets for managing liver diseases. Additionally, clinical trials are necessary to provide practical and economical approaches for managing liver diseases using herbal preparations that modulate the gut microbiota.

## Figures and Tables

**Table 1:** Population, Intervention, Comparison, and Outcome (PICO) framework.

PICO	Details
Population	Animal models
Intervention	Herbal preparations, either as a single herb or herbal mixture in any form of formulation
Comparator	Negative control
Outcome	Primary outcome: Effect on hepatobiliary system of herbal preparations Secondary outcome: Changes in liver enzyme levels Histological assessment of liver tissues Inflammation markers in the liver Lipid profile and metabolic parameters Gut microbiota composition analysis Oxidative stress markers Antioxidant enzyme activities Insulin resistance Bile acid Other relevant biochemical or molecular markers

**Table 2:** Inclusion and exclusion criteria.

Inclusion criteria	(a) Primary articles of in vivo (rodent) animal studies (b) Articles that investigated herbal preparations as an intervention in all types of formulations, including extracts, tablets, capsules, powders, liquids, decoctions, as a single herb, or a mixture of herbs (c) Articles that investigated the hepatoprotective mechanism through gut microbiota as the primary outcome
Exclusion criteria	(a) In vitro, in silico, and clinical studies (b) Review papers and book sections (c) Articles that investigated isolated compounds as the intervention (d) Compounds not isolated from herbal preparations or unmentioned sources (e) Any combination of herbs with other chemicals, such as sucrose, synthetic compounds, and prebiotics (f) Fermented herbal products (g) Partially or not accessible articles with abstract only and conference proceedings (h) Articles in languages other than English

**Table 3:** Summary of included studies using herbal preparations as hepatoprotective agents.

No.	Disease Models	Ref. Country	Dietary Models	Animal Model (Species; Gender)	Intervention Details (Herbs; Types of Extracts; Dose; Duration)	Comparator	Gut Microbiota Profiling Samples
1	NAFLD	[15] China	HFD	C57BL/6J mice; male	*Penthorum chinense* Pursh.; water extract; 2, 4, and 8 g/kg; 8 weeks	Untreated mice	Stool samples
2	NAFLD	[16] China	HFD	C57BL/6J mice; male	*Phyllanthus emblica*; aqueous extract; 0.9, 1.8, 3.6, and 6 g/kg; 6 weeks	Untreated mice	Stool samples
3	NAFLD	[17] China	HFD	Kunming mice; NS	*Polygoni Multiflori Radix Praeparata*; ethanol extract; 0.68, 1.35, 2.7, and 3 g/kg; 3 weeks	Untreated mice	Stool samples
4	NAFLD	[18] China	HFD	C57BL/6J mice; male	*Eriobotrya japonica*; NS; 25 and 50 mg/kg bw; 8 weeks	Oral gavage once daily, normal saline	Colonic mucosal samples
5	NAFLD	[19] China	HFD	SD rats; male	Walnut green husk; polyphenols; 100 and 200 mg/kg; 8 weeks	Untreated rats	Stool samples
6	NAFLD	[20] China	HFD	C57BL/6J mice; male	*Salviae miltiorrhizae*; polysaccharide; 10 and 20 mg/kg; 8 weeks	Untreated mice	Stool samples
7	NAFLD	[21] China	HFD	C57BL/6J mice; male	*Brassica juncea* var. tumida; water extract; 25 and 50 mg/kg; 8 weeks	Oral gavage once daily, normal saline	Colonic mucosal samples
8	NAFLD	[22] China	HFD	C57BL/6J mice; male	*Luffa cylindrica*; NS; 2 g/kg; 12 weeks	Oral gavage once daily, normal saline	Stool samples
9	NAFLD with low-grade colitis	[23] China	HFD	C57BL/6 mice; male	* Tetrastigma hemsleyanum * ; methanol; 100 and 500 mg/kg; 15 weeks	Untreated mice	Stool samples
10	NAFLD	[24] China	HFHC	C57BL/6 mice; male	Fufang Zhenzhu Tiaozhi (FTZ); ethanol–water extract; 12 g/kg BW/d; 7 weeks	Untreated mice	Stool samples
11	NAFLD	[25] China	HFD	C57BL/6J mice; male	Tianhuang Formula; ethanol extract; 100 mg/kg/day; 6 weeks	Untreated mice	Stool samples
12	Obesity, NAFLD	[26] China	HFD	SD rats; male	Walnut green husk; ethanol–water extract; 600 mg/kg BW; 50 days	Untreated rats	Stool samples
13	NAFLD	[27] China	HFFD	C3H mice; male	Yiqi-Bushen-Tiaozhi (YBT); NS; 26.76 g/kg/day; 12 weeks	Untreated mice	Stool samples
14	NAFLD	[28] China	HFHS	C57BL/6J mice; male	White peony root, licorice, grape seed, and broccoli mixture; NS; 22.5 mg/kg of licorice extract, 37.5 mg/kg of white peony extract, 15 mg/d of broccoli extract, and 30 mg/d of grape seed extract (low dose), double low dose (high dose); 14 weeks	Untreated mice	Stool samples
15	NAFLD	[29] China	HFD	C57BL/6 mice; male	Si Miao Formula (SMF); decoction; 10 and 20 mg/kg; 16 weeks	Oral gavage once daily, distilled water	Stool samples
16	NAFLD	[30] Japan	Western diet	C57BL/6N mice; male	*Vaccinium myrtillus* L.; anthocyanins extract; 2%; 12 weeks	Untreated mice	Stool samples
17	NAFLD	[31] Israel	HFD	C57BL/6J mice; male	*Cannabis sativa* L.; ethanol extract; 5 mg/kg BW; 8 weeks	Untreated mice	Stool samples
18	NAFLD	[32] Taiwan	HFD	Wistar rats; male	*Camellia oleifera*; hot water extract; 4.5 and 9 g/L; 8 weeks	Untreated rats	Stool samples
19	NAFLD	[33] China	HFD	SD rats; male	Da-Chai-Hu (DCH) decoction; aqueous extract; 8 g/kg; 8 weeks	Oral gavage once daily, normal saline	Stool samples
20	NAFLD	[34] China	HFD	SD rats; male	Dahuang Zexie decoction (DZD) herbal mixture; water extract; 5.13 g/kg; 4 weeks	Oral gavage once daily, normal saline	Stool samples
21	NAFLD	[35] China	HFD	SD rats; male	919 Syrup herbal mixture; 10 mL/kg; 4 weeks	Untreated rats	Stool samples
22	NAFLD	[36] China	HFD	C57BL/6J mice; male	*Morinda citrifolia* L.; ethanol–water extract; 100 and 200 mg/kg BW/d; 10 weeks	Untreated mice	Stool samples
23	NAFLD	[37] China	HFD	C57BL/6 mice; male	Jiang Zhi Granule; aqueous extract; 479 and 944 mg/kg/day; 8 weeks	Untreated mice	Stool samples
24	NAFLD	[38] China	HFD	C57BL/6/J mice; male	*Myristica fragrans* Houtt; ethanol-petroleum ether; 125 mg/kg; 4 weeks	Untreated mice	Stool samples
25	NAFLD	[39] China	HFD	SD rats; male	Jian-Gan-Xiao-Zhi decoction; water extract; 8 and 16 g/kg/bw; 12 weeks	Oral gavage once daily, normal saline	Stool samples
26	NAFLD	[40] China	HFD	Wistar rats; male	*Mallotus furetiamus*; NS; 1.7, 2.5 and 3.3 g/kg; 8 weeks	Untreated rats	Stool samples
27	NAFLD	[41] China	HFD	C57BL/6 mice; male	*Fagopyrum dibotrys*; ethanol extract; HFD mixed with FDE at a mass ratio of 9:1; 14 weeks	Untreated mice	Stool samples
28	NAFLD	[42] China	HFD	C57BL/6J mice; male	*Panax ginseng*; water extract; 100 and 200 mg/kg; 12 weeks	Untreated mice	Stool samples
29	NAFLD	[43] Japan	NS	ob/ob mice; male	Bofutsushosan (BTS) decoction; hot water extract; 5% BTS; 4 weeks	Untreated mice	Stool samples
30	NAFLD	[44] China	HFD	SD rats; male	Chinese Herbal Formula (CHF) (*Artemisia capillaries* Thunb., *Polygonum cuspidatum* Sieb. et Zucc, *Curcuma longa* L., *Hypericum japonicum*, *Gardenia jasminoides* Ellis) ethanol extract; 0.47 and 0.93 g/100 g body weight; 4 weeks	Untreated rats	Stool samples
31	NAFLD	[45] China	HFD	C57BL/6 mice; male	*Polygonatum cyrtonema* Hua; hot water extract; 200 and 400 mg/kg bw; 6 weeks	Untreated mice	Stool samples
32	NAFLD	[46] China	HFD with glucose and fructose	C57BL/6J mice; male	* Ficus hirta * Vahl. (FV); water extract; 5 and 10 g/kg BW; 17 weeks	Oral gavage once daily, normal saline	Stool samples
33	NAFLD	[47] China	HFD	C57BL/6J mice; male	*Pueraria lobata*; aqueous (PLS); 400 mg/kg bw; 8 weeks	Oral gavage once daily, distilled water	Stool samples
34	NAFLD	[48] China	HFD	Wistar rats; male	Qinghua Fang decoction (*Sedum sarmentosum* 15 g, *Radix salvia* 9 g, *Rhizoma atractylodis* macrocephalae 12 g, *Tangerine peel* 9 g, *Lotus leaf* 15 g, *Gynostemma pentaphyllum* 9 g, *Wolfiporia* *cocos* 9 g, *Eupatorium japonicum* 15 g, *Rhizoma* *alismatis* 15 g, and *Stigma maydis* 9 g); water extract; 0.80, 0.40, and 0.20 g/100 g body weight; 10 weeks	Oral gavage once daily, distilled water	Stool samples
35	NAFLD	[49] China	HFD	C57BL/6N mice; male	*Camellia sinensis*; ethanol extract; 50 and 100 mg/kg; 12 weeks	Oral gavage once daily, distilled water	Stool samples
36	NAFLD	[50] Japan	HFD	STAM mice; male	Daisaikoto (DST) [Bupleurum root (26.09%), Pinellia tuber (17.39%), Scutellaria root (13.04%), peony root (13.04%), Jujube (13.04%), immature orange (8.7%), rhubarb (4.35%), and ginger (4.35%)]; hot water extract; 3%; 3 weeks	Untreated mice	Stool samples
37	NAFLD	[51] Republic of Korea	HFD	C57BL/6J mice; male	Yijin-Tang (YTJ) decoction [crow-dipper (*Pinellia ternata* (Thunb.) Makino), 4 g sun-dried tangerine peel (*Citrus unshiu* Markovich), 4 g china root (*Poria cocos* F.A. Wolf), 2 g licorice (*Glycyrrhiza uralensis* Fisch. ex DC.), and 2 g ginger (*Zingiber officinale* Roscoe)]; hot water; 50 mg/kg and 100 mg/kg; 6 weeks	Untreated mice	Stool samples
38	NAFLD	[52] China	HFD	C57BL/6J mice; male	*Zanthoxylum bungeanum* Maxim; dried; 120 mg/kg b.w; 12 weeks	Untreated mice	Stool samples
39	NAFLD	[53] Brazil	HFD	C57BL/6J mice; male	*Myrciaria jaboticaba*, NS; 5%, 10%, and 15%; 4 weeks	Untreated mice	Stool samples
40	NAFLD	[54] China	HFD	C57BL/6J mice; male	Zhishi Daozhi decoction (912.8 g of *Citrus aurantium*, 6.4 g of *Rheum palmatum*, 19.2 g of *Coptis chinensis*, 12.8 g of *Scutellaria baicalensis*, 19.2 g of *Massa Medicata Fermentata*, 19.2 g of *Atractylodes macrocephala*, 19.2 g of *Poria cocos*, and 12.8 g of *Alisma orientate*); water extract; 14.5 g/kg/bw; 4 weeks	Untreated mice	Stool samples
41	NAFLD	[55] Japan	Western diet	C57BL/6N mice; male	*Ampelopsis grossedentata*; polyphenol; 0.5, 1 and 2%; 12 weeks	Untreated mice	Stool samples
42	NAFLD	[56] China	HFD	C57BL/6 mice; male	*Juglans regia* L.; ethanol extract; 0.6 and 1.2 g/kg; 12 weeks	Oral gavage once daily, saline	Stool samples
43	NAFLD	[57] China	Western diet	C57BL/6N mice; male	*Areca catechu:* areca nut extract (ANE), areca nut polyphenols (ANPs), and arecoline (ARE); 0.005% ARE, 0.25% ANPs and 0.5% ANE; 12 weeks	Untreated mice	Stool samples
44	NAFLD	[58] China	HFD	SD rats; male	Er-Chen decoction (15 g of *Pinellia ternata*, 15 g of *Citrus aurantium* L., 9 g of *Smilax glabra* Roxb., and 4.5 g of *Glycyrrhiza uralensis* Fisch.); water extract; 4.5 and 9 g/kg/d; 12 weeks	Oral gavage once daily, saline	Stool samples
45	NAFLD	[59] China	HFD	SD rats; male	*Gynostemma pentaphyllum* (GP) decoction; water extract; 1.5, 3, and 6 g/kg/d; 4 weeks	Oral gavage once daily, distilled water	Stool samples
46	NAFLD	[60] China	HFD	C57BL/6J mice; male	*Terminalia bellirica* (Gaertn.) Roxb; ethanol extract; 0.9, 1.8, and 3.6 g/kg; 14 weeks	Untreated mice	Stool samples
47	NASH	[61] China	HFD	SD rats; male	*Tylophora yunnanensis* Schltr; ethanol extracts, 80 and 160 mg/kg; 6 weeks	Untreated rats	Stool samples
48	NASH	[62] China	HFD	C57BL/6 J mice; male	*Citrus Varieties* (*Changshan Huyou*), flavonoids, 50 mg/kg; 12 weeks	Untreated mice	Stool samples
49	NASH	[63] China	MCD	C57BL/6 mice; male	Qian-Ghan decoction; water extract; 400 mg/kg; 4 weeks	Untreated mice	Stool samples
50	NASH	[64] China	MCD	C57BL/6 mice; male	Qingrequzhuo capsule (15 g of *Morus alba* L., 9 g of *Coptis chinensis* Franch., 12 g of *Anemarrhena asphodeloides* Bunge, 12 g of *Alisma plantago-aquatica* subsp. orientale (Sam.), 15 g of *Citrus aurantium* L., 9 g of *Carthamus tinctorius* L., 6 g of *Rheum palmatum* L., 15 g of *Smilax glabra* Roxb., 12 g of *Dioscorea oppositifolia* L., 12 g of *Cyathula officinalis*); water extract; 0.48, 0.96, and 1.92 g/kg; 6 weeks	Untreated mice	Stool samples
51	NASH	[65] China	MCD	C57BL/6 mice; male	*Salvia-Nelumbinis* naturalis; water–methanol extract; 750 mg/kg; 4 weeks	Untreated mice	Stool samples
52	Hepatic steatosis	[66] Mexico	HFD	C57BL/6J mice; male	*Agave salmiana;* saponin; 2.8 and 28 mg/kg; 8 weeks	Untreated mice	Stool samples
53	Hepatic steatosis	[67] China	HFD	C57BL/6J mice; male	*Malus domestica*; polyphenols; 125 and 500 mg/kg; 12 weeks	Untreated mice	Stool samples
54	Hepatic steatosis	[68] Canada	HFHS	C57BL/6J mice; male	Arctic berries; ethanol extract; 200 mg/kg; 8 weeks	Untreated mice	Stool samples
55	Hepatic steatosis	[69] China	HFFD	Kunming mice; male	*Artemisia sphaerocephala* Krasch; polysaccharides; 800 mg/kg; 8 weeks	Untreated mice	Stool samples

HFD = high-fat diet; HFHC = high-fat and high-cholesterol diet; HFFD = high-fat and high-fructose diet; HFHS = high-fat and high-sucrose diet; NS = not specified; MCD = methionine- and choline-deficient; SD = Sprague–Dawley; STAM = STelic Animal Model.

**Table 4:** Effects and mechanisms of herbal preparations compared with control on various liver diseases through gut microbiota modulation.

No.	Ref.	Herbs	Hepatoprotective Mechanisms/Outcomes						
			Liver Enzymes	Histopathological Parameters	Inflammation Markers	Lipid Profile	Antioxidant Enzyme Activity and Oxidative Stress Markers	Insulin Resistance/Bile Acid	Gut Microbiota Composition Analysis
1	[15]	*Penthorum chinense* Pursh.	Decreased AST and ALT	Lowered NAFLD score, reduced fatty droplets, and degenerated hepatocyte ballooning	NR	Decreased TC, TGs, and LDL-c	NR	NR/NR	Decreased F/B ratio
2	[16]	*Phyllanthus emblica*	Decreased AST and ALT	Ameliorated hepatocyte swelling/necrosis, inflammatory cell infiltration, and improved numerous red lipid droplets	NR	Decreased LDL-c, TGs, and HDL-c	NR	NR/NR	Decreased F/B ratio
3	[17]	*Polygoni Multiflori Radix Praeparata*	NR	Suppressed hepatocellular steatosis with clear hepatic cord structure, dilated hepatic sinusoids, and decreased degree of redness in tissues	NR	Inhibited TC, TG, and LDL-c rise; restored HDL-c	NR	NR/Recovered bile acids genes	Decreased F/B ratio
4	[18]	*Eriobotrya japonica*	NR	Alleviated abnormal situation of fat deposition	Lowered hepatic SREBP-1c and ChREBP	Inhibited TC and TG rise	(1) Decreased MDA level (2) Reduced activities of GSH-Px and T-SOD	NR/NR	Decreased F/B ratio
5	[19]	Walnut green husk	Inhibited an abnormal increase in AST and ALT	Prevented abnormal situation of liver fat deposition	(1) Reduced IL-1β, IL-6, TNF-α, and MCP-1 (2) Upregulated the expression of Nrf2 (3) Suppressed upregulation of the mRNA expression of colonic IL-1β, IL-6, and TNF-α	(1) Inhibited TC, TG, LDL-c, and NEFA rise (2) Prevented HDL-c decrease	Lowered GSH-Px and T-SOD in liver	NR/NR	(1) Decreased F/B ratio and reversed *Ruminococcaceae-UCG-014* and *Fusicatenibacter* (2) Increased expression levels of ZO-1, occludin, and MUC-2 mRNA in the colon
6	[20]	*Salviae miltiorrhizae*	Decreased ALT and AST	(1) Displayed much more normal hepatic histological morphology and steatosis grade (2) Decreased lipid droplets (3) Induced collagen deposition	(1) Suppressed hepatic expressions of TIMP1 and α-SMA (2) Reversed the decreased trend in the expressions of anti-inflammatory genes (IL-2, IL-10, TGF-B) (3) Reversed the increased trend in the expressions of pro-inflammatory genes (IL-6 and IL-23)	Decreased HDL-c, LDL-c, TGs, TC, NEFAs, and FBG	NR	NR/NR	Reversed increase in *Bifidobacterium* and *Lactobacillus*
7	[21]	*Brassica juncea var. tumida Tsen et Lee*	Decreased AST and ALT	Prevented dyslipidemia, oxidative stress, and hepatocyte damage by reducing hepatic vacuole and gap	Regulated the FOXO, MAPK, and the p53 signaling pathways	(1) Prevented TG and LDL-c increase; maintained HDL-c (2) Decreased hepatic fat	Increased GSH	NR/NR	Increased Bacteroidetes and Firmicutes
8	[22]	*Luffa cylindrica*	Decreased ALT and AST	Ameliorated lipid accumulation in hepatocytes and inhibited development of steatosis	Reduced MCP-1 expression in liver	Decreased TGs, TC, LDL, and FFAs	NR	Decreased fasting insulin in serum and enhanced insulin sensitivity/NR	NR
9	[23]	* Tetrastigma hemsleyanum *	Decreased AST	Relieved symptoms of damaged liver structure	(1) Decreased IL-6, TNF-α (2) Lowered MCP-1	(1) Increased HDL-c; decreased LDL-c (2) Decreased PPAR-gamma relative expression (3) Decreased ACC-1 and SREBP-1c genes	(1) Increased SOD; decreased MDA (2) Increased Nrf2 expression (3) Upregulated NQO1 and HO-1 (4) Expressed NQO1, GCLC, and HO-1 genes	(1) Improved glucose clearance rate (2) Decreased serum TBA/NR	(1) Increased SCFAs (2) Increased gut microbiota abundance (3) Increased phylum level of Bacteroidetes, Firmicutes, and *Verrucomicrobia* (4) Enriched *Akkermansia* and Lanchnospiraceae (5) Upregulated occludin and ZO-1 expression
10	[24]	Fufang Zhenzhu Tiaozhi (FTZ)	Decreased ALT and AST	Ameliorated extent of steatosis and lipid droplet numbers in liver	NR	Decreased TC and TGs	NR	NR/NR	Increased the abundance of *Bacteroidetes* and reduced the ratio of *Firmicutes*/*Bacteroidales*
11	[25]	Tianhuang Formula	Decreased ALT and AST	Reduced hepatic steatosis and lipid accumulation	NR	Reduced TC, TGs, and LDL-c	(1) Increased GSH and SOD levels while decreasing MDA level (2) Increased mRNA expression of hepatic TRX1, GCLC, and NQO1 (3) Increased protein expressions of Nrf2, NQO1, and HO-1 (4) Decreased Keap1 protein expression	NR/NR	Increased *Lactobacillus* and its metabolites 5-MIAA
12	[26]	Walnut green husk	Decreased ALT and AST	(1) Reduced liver weight (2) Reduced adipocyte size (3) Improved abnormal liver lipid accumulation	Decreased IL-1β, IL-6, TNF-α, and MCP-1	Reduced TC, TGs, and LDL-c	(1) Improved liver MDA level (2) Decreased P-JNK/JNK ratio (3) Increased Nrf2 level (4) Enhanced activities of GSH-Px and T-SOD (5) Inhibited liver PPARα increase	NR/NR	(1) Increased bacterial generation of SCFAs (*Allobaculum*, *Prevotellaceae*, etc.) (2) Decreased F/B ratio (3) Reduced Lactobacillaceae and Lachnospiraceae
13	[27]	Yiqi-Bushen-Tiaozhi (YBT)	Decreased ALT and AST	(1) Improved hepatic inflammation and steatosis (2) Reduced staining of collagen fibers to varying degrees	NR	Improved TGs, TC, HDL-c, and LDL-c	NR	NR/NR	Increased *Rikenellaceae*, *Erysipelotrichaceae_ incertae_sedis*, and *Clostridium_XIVb*
14	[28]	White peony root, licorice, grape seed, broccoli	Decreased ALT and AST	Increased liver weight and hepatic fat deposition	Reduced LPS serum level	Reduced TC, TGs, and LDL-c	(1) Downregulated mRNA expression of TLR2, TLR4, and TLR9 (2) Reduced p-JNK/JNK ratio (3) Suppressed inflammatory pathways (MCP-1, ICAM-1, Adcy7, Dock2, Ccr2, Cxcl14, Cx3cr1, Mif, IL-6, and Ccl7)	NR/NR	(1) Increased *Ruminiclostridium_9* and *Blautia* (2) Reduced *Anaerotruncus*, *Bacteroides_Otu199*, *Desulfovibrio* genera (3) Reduced mRNA and protein expression of ZO-1 and occludin
15	[29]	Si Miao Formula (SMF)	Decreased ALT and AST	(1) Decreased adipocyte volume (2) Inhibited macrovascular steatosis	(1) Inhibited Nlrp-3 and IL-1α expression (2) Reduced Mcp-1 in eWAT expression	Decreased TC, LDL-c, and TGs	NR	(1) Reduced FBG and AUC values of IPGTT and IPITT (2) Upregulated glucose metabolism-related pathways/NR	(1) Increased *Akkermansia* genus within Verrumicrobia phylum and *Faecalibaculum* and *Caproiciproducens* genus (2) Decreased Firmicutes
16	[30]	*Vaccinium myrtillus* L.	Decreased AST and ALT	(1) Decreased final body weight, liver weight, liver weight/body weight ratio, and liver fat weight and content (2) Decreased liver lipid droplet accumulation	Reduced MCP-1	Decreased TC and LDL-c	(1) Decreased TBARS, α-SMA, and Keap-1 (2) Increased SOD2, Nrf2, and ubiquitinated Nrf2 (Ub-Nrf2)	Reduced insulin levels and insulin resistance/NR	(1) Decreased F/B ratio and *Deferribactere*, S24-7, *Prevotella*, *Lactobacillales*, and *Clostridiales* abundance (2) Increased *Verrucomicrobia*, *Bacteroides*, *acidifaciens*, *Parabacteroides*, *Akkermansia*, and *muciniphila*
17	[31]	*Cannabis sativa* L.	NR	Decreased adipose tissue weight, severity of centrilobular hepatocytic vacuolation (fatty change)	NR	NR	NR	NR/NR	(1) Decreased Firmicutes Bacteroidetes (F/B) ratio, Bacteroidetes, and *Betaproteobacteria* abundance (2) Increased Firmicutes, *Clostridia*, *Flavobacteria*, Deferribacteraceae, *Bifidobacterium*, *Prevotella*, *Lactobacillus*, and *Akkermansia muciniphila*
18	[32]	*Camellia oleifera*	Decreased ALT and AST	(1) Lowered weight gain, abdominal fat, and liver weights (2) Suppressed hepatic fatty change and inflammation	Lowered TNF-α	Lowered TC and TAG concentrations	Lowered MDA	Lowered AUC of OGTT, FBG, and HOMA-IR levels/NR	Increased anaerobe/aerobe ratio
19	[33]	DCH decoction	Decreased liver index, serum AST, and ALT	(1) Decreased body weight, NAS score, lipid-loaded hepatocytes (2) Improved hepatic steatosis, hepatocyte ballooning, and lobular inflammation	NR	Decreased TGs and TC	(1) Increased SOD and GSH-Px (2) Decreased MDA	Lowered AUC of OGTT, FINS, and HOMA-IR levels/NR	(1) Increased *Romboutsia*, *Bacteroides*, *Lactobacillus*, *Akkermansia*, and *Turicibacter* (2) Decreased *Lachnoclostridium* and Enterobacteriaceae (3) Decreased F/B ratio
20	[34]	DZD herbal mixture	Decreased ALT and AST	(1) Decreased body weight and liver weight (2) Relieved macrosteatosis, hepatocyte ballooning	NR	Decreased TGs, TC, HDL-C, and LDL-C	NR	NR/NR	(1) Decreased Firmicutes, Desulfovibrionaceae, *Desulfovibrio*, and *Escherichia/Shigella* (2) Increased *Bacteroidetes*, *Ruminococcaceae*, *Bacteroides*, *Oscillibacter*, and *Butyricicoccus*
21	[35]	919 Syrup herbal mixture	NR	NR	NR	NR	NR	NR/NR	(1) Increased Firmicutes, *Proteobacteria*, *Actinobacteria*, *Tenericutes*, *Lactobacillus*, and Ruminococcaceae_UCG-005 ratios (2) Reduced Bacteroidetes, *Spirochaetes*, *Cyanobacteria*, and *Epsilonbacteraeota* ratios
22	[36]	*Morinda citrifolia* L.	Suppressed ALT and AST	(1) Normalized body weight, liver index, and fat index (2) Reduced white adipose tissue (WAT) adipocyte sizes (3) Alleviated liver injury	(1) Decreased serum LPS, TNF-α, IL-β, IL-6, TLR4, MyD88, and NF-κB p65 (2) Improved liver inflammation by suppressing LPS/TLR4/NF-κB pathway	(1) Decreased TC, TGs, and LDL-c (2) Increased HDL-c	(1) Reduced MDA (2) Increased GSH and CAT activity	(1) Decreased FBG and FINS levels (2) Decreased blood glucose, AUC of OGTT, and HOMA-IR levels/NR	(1) Increased Bacteroidota and decreased Firmicutes, *Campilobacterota*, *Desulfobacterota*, *Proteobacteria*, *Parabacteroides*, *Prevotellaceae_UCG-001*, *Lactobacillus*, *Roseburia*, *Akkermansia* genera, *Akkermansia muciniphila*, and *Bacteroides acidifaciens* (2) Decreased F/B ratio
23	[37]	Jiang Zhi Granule	Decreased ALT	(1) Reduced liver lipid deposition (2) Ameliorated extensive micro/macrovascular steatosis and frequent incidence of hepatocyte ballooning	NR	Decreased TC, TGs, and FFAs	Decreased MDA	Decreased serum insulin and HOMA-IR levels/NR	(1) Decreased proteobacteria and F/B ratio (2) Decreased Ruminococcaceae, Desulfovibrionaceae, Rikenellaceae, Dehalobacteriaceae, Christensenellaceae, Peptococcaceae (3) Increased S24_7 and Lachnospiraceae
24	[38]	*Myristica fragrans* Houtt (nutmeg)	Decreased AST and ALT	Reduced lipid accumulation	Reduced TNF-α, IL-6, and IL-1β	Decreased LDL-c, TC, and TGs	NR	NR/NR	(1) Regulated Bacteroidetes and Firmicutes (2) Increased probiotic species such as *Lactobacillus*, *Akkermansia*, and Bacteroides
25	[39]	Jian-Gan-Xiao-Zhi decoction	Decreased AST and ALT	Alleviated hepatocyte steatosis	Decreased IL-6, IL-1β, and TNF-α	Decreased TGs and TC	NR	NR/NR	(1) Decreased F/B ratio (2) Increased probiotics such as *Alloprevotella*, *Lactobacillus,* and *Turicibacter* (3) Increased ZO-1 and occludin
26	[40]	*Mallotus furetiamus*	Decreased AST, ALT, and GGT	Reduced liver steatosis	Reduced IL-1β, IL-6, TNFa, LEP, and ADP levels	Reduced TC, TGs, LDL-c, and HDL-c	NR	NR/NR	Increased *Bacteroides fragilis*, *Escherichia coli,* and *Staphylococcus xylosus*
27	[41]	*Fagopyrum dibotrys*	Decreased ALT, AST, and ALP	Reduced hepatic steatosis and lipid accumulation	NR	(1) Reduced TGs, TC, and LDL (2) Increased HDL	NR	Reduced AUCs of the IGT and IIT tests/NR	(1) Alleviated microbial community richness (2) Increased Bacteroidetes and *Verrucomicrobia* population (3) Decreased Firmicutes population and increased *Akkermansia*
28	[42]	Panax ginseng	Decreased AST and ALT	Decreased hepatic accumulation of lipids	Reduced TNF-α, IL-1β, and IL-6	Reduced TC, TGs, LDL-c, and LDL-C/HDL-c ratio	NR	NR/NR	(1) Increased richness and diversity of gut microbiota (2) Increased Bacteroidetes and decreased F/B ratio
29	[43]	Bofutsushosan (BTS) decoction	Decreased AST and ALT	Reduced cellular lipid accumulation, hepatocyte ballooning, and accumulation of inflammatory cells	NR	Decreased TC	NR	NR/NR	Increased *Bacteroides*, *Akkermansia,* and Enterobacteriaceae and decreased *Prevotella*
30	[44]	CHF	NR	(1) Reduced fat deposition in hepatocytes (2) Reduced liver index	NR	Decreased TGs and FFAs	NR	NR/NR	(1) Reduced intensities of *Coprococcus catus GD/7*, *Ruminococcus gauvreauii* strain, and *Escherichia coli* strain CAIM 1647 (2) Increased *Actinobacteria*
31	[45]	*Polygonatum cyrtonema* Hua	Decreased AST and ALT	Reduced liver weight, vacuolation, fat accumulation, and liver damage	NR	Decreased TGs, TC, and LDL-c; increased HDL-c	Increased SOD and GSH; decreased MDA	Enhanced glucose regulation/NR	(1) Improved species richness and diversity of bacterial community (2) Reduced F/B ratio (3) Increased *Allobaculum*, *Ruminococcus,* and *Bifidobacterium* and decreased *Helicobacter* and *Acinebacter* (4) Ameliorated decrease in SCFAs, such as isobutyrate and isovalerate content, and promoted production of acetate, propionate, and valerate
32	[46]	* Ficus hirta * Vahl. (FV)	NR	(1) Reduced liver injury (2) Decreased accumulation of lipid droplets in hepatocytes	(1) Reduced the mRNA expression of Srebp-1, Acaca, Hmgcr, Fabp1, Pparγ, and Cd36 (2) Increased ACOX1 and CPT1α levels (3) Downregulated the levels of IL-6, IL-1β, and TNF-α in serum (4) Suppressed the upregulated proteins of TNF-α, IL-6, and IL-1β in the liver tissues (5) Suppressed the mRNA expression of Tnf-α, IL-1β, and Ccl5 in liver tissues	(1) Decreased TGs and TC in liver (2) Decreased TC and LDL-c in serum	NR	NR/NR	(1) Reduced F/B ratio (2) Produced SCFAs
33	[47]	*Pueraria lobata*	Decreased AST and ALT	(1) Attenuated hepatic ballooning, inflammation, and fibrosis (2) Decreased histological scores	(1) Decreased IL-6 and TNF-alpha	Reduced TC, TGs, and LDL-c	NR	NR/NR	(1) Decreased Firmicutes (2) Increased Bacteroidota
34	[48]	Qinghua Fang decoction	NR	Improved liver injury	Reduced TNF-α, IL-6, IL-8, and IL-17 indexes	NR	NR	NR/NR	Increased richness and diversity of gut microbiota
35	[49]	* Camellia sinensis *	Decreased ALT, AST, and AKP	(1) Reduced, scattered, and made sparse the number of lipid droplets in liver cells (2) Alleviated fat lesions and the volume of lipid droplets	(1) Decreased IL-1β, IL-4, IL-6, IL-10, TNF-α, and IFN-γ	(1) Decreased D-LA, DAO, and LPS (2) Decreased TC, TGs, and LDL-c (3) Increased HDL-c	(1) Reduced ROS in liver tissue	NR/NR	(1) Decreased Firmicutes in feces (2) Increased *Bacteroides* and *Akkermansia* (3) Decreased F/B ratio
36	[50]	DST	NR	(1) Improved hepatic steatosis (2) Decreased hepatic ballooning	NR	(1) Decreased arachidonic acid, 13,14-dihydro-15-keto prostaglandin J2, and prostaglandin F2α (2) Decreased lipid mediators (derived from omega-3 fatty acids and omega-6 fatty acids)	NR	NR/NR	(1) Improved richness and evenness of microbiome (2) Improved percentage of microbiome composition at family level
37	[51]	YTJ decoction	Reduced AST, ALT	Decreased hepatic steatosis and liver damage	NR	NR	NR	NR/NR	Increased *B. acidifaciens*
38	[52]	*Zanthoxylum bungeanum* Maxim	NR	(1) Improve lipid deposition (2) Relieved lipid accumulation	NR	Decreased TC, TGs, and LDL-c	NR	NR/NR	(1) Increased *Bacteroidota* (2) Decreased *Actinobacteria* (3) Decreased harmful microflora related to metabolic diseases (4) Increased beneficial bacteria
39	[53]	*Myrciaria jaboticaba*	Decreased AST and ALT	(1) Reduced lipid droplets (2) Completely reversed steatosis (3) Reduced hepatic nuclear binucleation	Decreased IL-6 and TNF-α	Reduced TC and LDL-c	NR	(1) Decreased glucose level and HOMA-IR index values (2) Lowered insulin level/NR	(1) Lowered bacteria number (2) Decreased F/B ratio (3) Decreased richness and abundance of bacteria community composition (4) Increased Lachnospiraceae and Enterobacteriaceae families and *Parabacteroides*, *Sutterella*, *Allobaculum*, and *Akkermansia* genera
40	[54]	Zhishi Daozhi decoction	Decreased AST and ALT	(1) Alleviated the arrangement of hepatocytes, fat vacuoles of varying sizes, and the marginalized nuclei (2) Decreased irregular cell shape with unclear border, marginalized nuclei, and lipid droplets of different sizes	NR	Reduced TGs and TC	NR	NR/Maintained high levels of acetic acid (AA), propionic acid (PA), butyric acid, and SCFAs	(1) Decreased trend in community richness (2) Altered intestinal flora (3) Decreased Firmicutes, increased Bacteroidetes (4) Reduced F/B ratio
41	[55]	*Ampelopsis grossedentata*, vine tea polyphenol (VTP)	Decreased AST and ALT	(1) Decreased WD-induced body weight, liver weight, and epididymal fat weight (2) Reduced lipid droplets and prevented hepatocyte ballooning degeneration in a dose-dependent manner	NR	(1) Reduced TGs, TC, LDL, and VLDL (2) Reduced hepatic lipids content, hepatic TGs, and TC content	(1) Increased hepatic Nrf2 level and target enzymes of Nrf2, such as NQO1 and HO-1 (2) Decreased level of hepatic TBARS, a byproduct of lipid peroxidation	Decreased the serum insulin level/NR	Increased *Akkermansia* and reduced F/B ratio
42	[56]	*Juglans regia* L., defatted walnut powder extract (DWPE)	NR	(1) Lowered liver weights and Lee’s index (2) Decreased lipid droplets and hepatocyte ballooning	Decreased expressions of proteins related to inflammation (p-p38, p-ERK, p-JNK, and p-NF-κB) in the liver	Decreased TGs, TC, and LDL; increased HDL	Reduced MDA level	NR/NR	(1) Reverted the profiles of gut microbiota (2) Decreased *Erysipelotrichia*, Firmicutes, and *Actinobacteria* while increased *Clostridiales*, *Bacteroidales*, Prevotellaceae, and *Bacteroides*
43	[57]	Areca catechu (ANE, ANPs, and ARE)	Decreased AST, ALT, and ALT/AST ratio	Decreased liver weight and epididymal fat	NR	(1) Reduced liver’s total lipid rate (2) Reduced lipid droplets (3) Reduced T-Cho, HDL, and non-HDL (4) Activated p-AMPKα and decreased expression levels of SREBP2 and HMGCR	NR	NR/NR	Increased *Akkermansia* and decreased *Ruminococcus*
44	[58]	Er-Chen decoction	Decreased TC, TGs, ALT, and AST	Alleviated steatosis and inflammatory cell infiltration and decreased lipid accumulation	Reduced IL-6 and IL-1β and mRNA expression of TNF-α	NR	Enhanced activities of SOD and GSH-Px and decreased MDA levels	NR/NR	Increased *Lactobacillus*, *Dubosiella*, Lachnospiraceae, *Akkermansia*, and *Intestinimonas* and decreased *Desulfovibrio* and *C._saccharimonas*
45	[59]	*Gynostemma pentaphyllum* (GP)	Decreased AST and ALT	Reduced the extent of steatosis	Decreased TNF-α, IL-1β, and IL-6	Decreased TC, TGs, and LDL-c; increased HDL	NR	Reduced endotoxemia and insulin resistance/NR	(1) Decreased F/B ratio (2) Increased beneficial bacteria, such as *Lactococcus* spp., and decreased pathogenic bacteria, including *Ruminococcus* spp.
46	[60]	*Terminalia bellirica* (Gaertn.) Roxb	Decreased AST and ALT	Attenuated lipid accumulation and hepatocellular necrosis	NR	Reduced TGs and LDL-c; increased HDL-c	NR	NR/NR	(1) Restored structural disturbances of gut microbiota (2) Reduced *Intestinimonas*, *Lachnoclostridium*, and *Lachnospirace-ae_FCS020_*group and increased *Akkermansia* and *Bifidobacterium*
47	[61]	*Tylophora yunnanensis* Schltr	Decreased AST and ALT	Reduced infiltration of lipid droplets and hepatic steatosis	Decreased the protein expression levels of LPS, FFAs, VLDL, IL-6, IL-1β, TNF-α, TGF-β, caspase-1, ASC, and NLRP3 in the liver	(1) Decreased TC, TGs, and LDL-c; increased HDL-c	NR	NR/NR	(1) Increased *Lachnospiraceae*, *Muribaculaceae*, *Peptostreptococcaceae*, *Christenellaceae*, and *Coriobacteriaceae* (2) Inhibited NLRP3, thereby reversing composition of gut microbiota to improve NASH
48	[62]	*Citrus Varieties* (*Changshan Huyou*)	Decreased ALT	Reduced degree of fatty, infiltration of inflammatory cells, and hepatocyte ballooning	NR	NR	NR	NR/NR	(1) Decreased Chao1, ACE (2) Increased *Christensenellaceace*, *Allobacullum,* and *Erysipelotrichaceae* (3) Decreased *Porphyromonadaceae*, *Streptococcaceae*, and *Eubacterium* and decreased *Porphyromonadaceae*
49	[63]	Qian-Ghan decoction	Decreased AST and ALT	Decreased infiltration of inflammatory cells and NAS value in liver sections	(1) Decreased the mRNA expression of TNF-α and IL-β and expression of TLR4, MYD88, and NF-ĸB (2) Decreased the protein expression of TLR4 and reduced the phosphorylation of IKBα and P65 in MCD diet-induced NASH mice	Decreased TGs and TC	NR	NR/(1) Decreased serum and liver BA concentration (2) Decreased serum TDCA and TLCA levels (3) Reversed liver CA increase but had no effect on liver βMCA and GCA, TCA, and TβMCA levels (4) Increased fecal levels of secondary BAs, such as α + ω MCA and LCA	Increased *Clostridia* and *Bacteroides*
50	[64]	Qingrequzhuo capsule	Decreased AST and ALT	Alleviated disorganized hepatic plate arrangement, inflammatory cell infiltration, and a large amount of vacuolar-like degeneration; reduced hepatic steatosis and reduced fat droplets	Decreased IL-1β, IL-6, and TNF-α	Decreased TC and TGs	Decreased MDA; increased SOD and GSH-Px activities	NR/NR	(1) Increased diversity of gut microbiota and affected relative abundances of *Dubosiella* and *Blautia* (2) Decreased Firmicutes, Bacteroidetes and *Lachnospiraceae_NK4A136_group*
51	[65]	*Salvia-Nelumbinis* naturalis	Decreased ALT	Reduced liver steatosis, inflammation, ballooning, and total NAS score	(1) Decreased mRNA expression of SREBP1, ACC, FASN, and SCD1 (2) Decreased ratio of phosphorylated p65 to total p65 (3) Decreased mRNA expression of inflammatory TNF-α and IL-6	Decreased TGs	NR	NR/ Decreased TBA level, increased fecal norDCA and norCA, and decreased GHCA, wMCA, TwMCA, GCA, and GDCA levels	(1) Decreased F/B ratio (2) Decreased enrichment of *Coriobacteriaceae UCG-002*, *Corynebacterium 1*, *Clostridium sensu stricto 1,* and *Glutamicibacter*, whereas increased *Romboutsia*, *Ruminiclostridium 9*, *Alloprevotella*, *Candidatus Saccharimonas*, *Roseburia*, *Lachnospiraceae NK4A136 group,* and *Akkermansia*
52	[66]	*Agave salmiana*	Increased ALT	Decreased hepatic lipid accumulation	Increased TNF-α	Lowered LDL-c	Decreased MDA	Lowered insulin concentrations/NR	Increased *Akkermansia muciniphila*
53	[67]	Apple polyphenols	NR	NR	NR	NR	NR	NR/NR	(1) Increased *Akkermansia* and *Faecalibaculum* (2) Decreased *Enterococcus* and *Lactobacillus*
54	[68]	Arctic berry extracts	NR	NR	Lowered the mRNA expression of Tlr4 and liver inflammation	(1) Reduced hepatic triacylglycerol accumulation and triacylglycerolemia (2) Increased lipid oxidation	NR	NR/Increased tyrosine-phosphorylated CEACAM-1	(1) Decreased F/B ratio (2) Increased presence of *Peptostreptococcaceae*, *Akkermansia muciniphila,* and *Turicibacter* and lower presence of *Lactobacillus* and *Bifidobacterium* in fecal microbiota (3) Increased *Oscillibacter* and *Turicibacter* (4) Increased branched-chain SCFAs in feces
55	[69]	*Artemisia sphaerocephala* Krasch	NR	(1) Exhibited less microvesicular fatty changes and reduced adipocyte size (2) Reduced number and volume of hepatic fat granules	NR	(1) Decreased hepatic C16:1 and C18:1 levels, TC, TGs, and NEFAs (2) Lowered hepatic ACC, FAS, and SCD-1 and decreased expression of proteins involved in lipogenesis	NR	NR/NR	(1) Decreased Firmicutes (2) Increased Bacteroidetes and *Akkermansia*

NR: Not reported; AST: aspartate aminotransferase; ALT: alanine aminotransferase; TC: total cholesterol; TG: triglycerides; LDL-c: low-density lipoprotein cholesterol; F/B: Firmicutes/Bacteroidetes ratio; HDL-C: High-Density Lipoprotein Cholesterol; SREBP-1c: Sterol Regulatory Element-Binding Protein 1c; ChREBP: Carbohydrate-Responsive Element-Binding Protein; MDA: Malondialdehyde; GSH-Px: Glutathione Peroxidase; T-SOD: Total Superoxide Dismutase; IL-1β: Interleukin 1 Beta; IL-6: Interleukin 6; TNF-α: Tumor Necrosis Factor Alpha; MCP-1: monocyte chemoattractant protein 1; Nrf2: Nuclear Factor Erythroid 2-related Factor 2; NEFAs: Non-Esterified Fatty Acids; ZO-1: zonula occludens-1; MUC-2: Mucin 2; TIMP1: Tissue Inhibitor of Metalloproteinase 1; α-SMA: Alpha-Smooth Muscle Actin; IL-2: Interleukin 2; IL-10: Interleukin 10; TGF-β: Transforming Growth Factor Beta; FBG: fasting blood glucose; FOXO: Forkhead Box Protein O; MAPK: Mitogen-Activated Protein Kinase; p53: Tumor Protein 53; FFAs: free fatty acids; PPAR-gamma: Peroxisome Proliferator-Activated Receptor Gamma; ACC-1: Acetyl-CoA Carboxylase 1; NQO1: NAD(P)H Quinone Dehydrogenase 1; HO-1: Heme Oxygenase-1; GCLC: Glutamate-Cysteine Ligase Catalytic Subunit; TBA: total bile acid; SCFAs: short-chain fatty acids; TRX1: Thioredoxin 1; Keap1: Kelch-Like ECH-Associated Protein 1; 5-MIAA: 5-Methylthioadenosine; ICAM-1: Intercellular Adhesion Molecule 1; Adcy7: Adenylate Cyclase 7; Dock2: Dedicator of Cytokinesis 2; Ccr2: CC Chemokine Receptor 2; Cxcl14: C-X-C Motif Chemokine Ligand 14; Cx3cr1: C-X3-C Motif Chemokine Receptor 1; Mif: Macrophage Migration Inhibitory Factor; Ccl7: C-C Motif Chemokine Ligand 7; P-JNK/JNK: Phosphorylated c-Jun N-terminal Kinase (JNK); LPS: lipopolysaccharide; Nlrp-3: Nucleotide-binding oligomerization domain, Leucine rich Repeat and Pyrin domain containing 3; IPGTT: intraperitoneal glucose tolerance test; IPITT: intraperitoneal insulin tolerance test; TBARS: Thiobarbituric Acid Reactive Substances; TAG: Triacylglycerol; AUC: Area Under the Curve; OGTT: Oral Glucose Tolerance Test; HOMA-IR: Homeostatic Model Assessment for Insulin Resistance; FINS: Fasting Insulin; WAT: White Adipose Tissue; MyD88: Myeloid Differentiation Primary Response 88; NF-κB p65: nuclear factor kappa B p65 subunit; TLR4: Toll-like receptor 4; CAT: Catalase; GGT: gamma-glutamyl transferase; LEP: Leptin; ADP: Adenosine Diphosphate; Acaca: Acetyl-CoA Carboxylase Alpha; Hmgcr: 3-Hydroxy-3-Methylglutaryl-CoA Reductase; Fabp1: Fatty Acid-Binding Protein 1; Pparγ: Peroxisome Proliferator-Activated Receptor Gamma; Cd36: Cluster of Differentiation 36; ACOX1: Acyl-CoA Oxidase 1; Ccl5: Chemokine (C-C Motif) Ligand 5; AKP: alkaline phosphatase; IFN-γ: Interferon Gamma; D-LA: D-lactic Acid; DAO: Diamine Oxidase; AA: Arachidonic Acid; PA: Palmitic Acid; BA: Bile Acid; VLDL: very low-density lipoprotein; p-p38: Phosphorylated p38 (protein kinase); p-ERK: Phosphorylated Extracellular Signal-Regulated Kinase; p-JNK: Phosphorylated c-Jun N-terminal Kinase; p-NF-κB: Phosphorylated Nuclear Factor Kappa B.

## Supplementary Materials

The following supporting information can be downloaded at: https://www.mdpi.com/article/10.3390/cimb46100682/s1, Table S1: Search strategy and keywords.
