# Supplementary material for: A Scoping Review on Hepatoprotective Mechanism of Herbal Preparations through Gut Microbiota Modulation
Source: Curr Issues Mol Biol. 2024 Oct 16;46(10):11460–502. doi: 10.3390/cimb46100682 (PMC11506797; doi:10.3390/cimb46100682)
Supplement: Supplementary file 1 [file cimb-46-00682-s001.zip › Supplementary Materials Table S1 PDF.pdf]

Table S1: Search strategy and keywords.

1. **Search strategy**

   #1 (herbal medicine*) OR (medicinal plant*) OR (medicinal herb*) OR (herbal product*) OR (herbal plant*) OR (herbal extract*) OR (plant extract*) OR (phytotherap*) OR (botanical drug*) OR (nutraceutical*) OR (phytopharmaceutical*) OR (natural product*) OR (phytoconstituent*) OR (phytochemical*)

   #2 (hepatoprotect*) OR (liver protect*) OR (liver disease*) OR (cirrhosis) OR (liver damage*) OR (liver injur*) OR (hepatotox*)

   #3 (gut microbiota) OR (gut microbiome) OR (gut bacteria) OR (gut flora) OR (gut axis) OR (gastrointestinal microbiota) OR (gastrointestinal microbiome)

   #1 AND #2 AND #3

2. **Search results**
   Database searched on 2nd June 2023: PubMed 76 studies, Scopus 232 studies, EMBASE 69 studies, and Web of Science 1464 studies.

PubMed

Set	Keywords	Results
**1**	((((((((((((herbal medicine*) OR (medicinal plant*)) OR (medicinal herb*)) OR (herbal product*)) OR (herbal plant*)) OR (herbal extract*)) OR (plant extract*)) OR (phytotherap*)) OR (botanical drug*)) OR (nutraceutical*)) OR (phytopharmaceutical*)) OR (natural product*)) OR (phytoconstituent*)) OR (phytochemical*)	157,357
**2**	((((((hepatoprotect*) OR (liver protect*)) OR (liver disease*)) OR (cirrhosis)) OR (liver damage*)) OR (liver injur*)) OR (hepatotox*)	274,620
**3**	((((((gut microbiota) OR (gut microbiome)) OR (gut bacteria)) OR (gut flora)) OR (gut axis)) OR (gastrointestinal microbiota)) OR (gastrointestinal microbiome)	55,791
**4**	((((((((((((((herbal medicine*) OR (medicinal plant*)) OR (medicinal herb*)) OR (herbal product*)) OR (herbal plant*)) OR (herbal extract*)) OR (plant extract*)) OR (phytotherap*)) OR (botanical drug*)) OR (nutraceutical*)) OR (phytopharmaceutical*)) OR (natural product*)) OR (phytoconstituent*)) OR (phytochemical*)) AND (((((((hepatoprotect*) OR (liver protect*)) OR (liver disease*)) OR (cirrhosis)) OR (liver damage*)) OR (liver injur*)) OR (hepatotox*))) AND (((((((gut microbiota) OR (t microbiome)) OR (gut bacteria)) OR (gut flora)) OR (gut axis)) OR (gastrointestinal microbiota)) OR (gastrointestinal microbiome))	76

Scopus

Set	Keywords	Results
**1**	(((((((((((((herbal medicine*) OR (medicinal plant*)) OR (medicinal herb*)) OR (herbal product*)) OR (herbal plant*)) OR (herbal extract*)) OR (plant extract*)) OR (phytotherap*)) OR (botanical drug*)) OR (nutraceutical*)) OR (phytopharmaceutical*)) OR (natural product*)) OR (phytoconstituent*)) OR (phytochemical*)	313,584
**2**	((((((hepatoprotect*) OR (liver protect*)) OR (liver disease*)) OR (cirrhosis)) OR (liver damage*)) OR (liver injur*)) OR (hepatotox*)	215,591
**3**	((((((gut microbiota) OR (gut microbiome)) OR (gut bacteria)) OR (gut flora)) OR (gut axis)) OR (gastrointestinal microbiota)) OR (gastrointestinal microbiome)	70,230
**4**	((((((((((((((herbal medicine*) OR (medicinal plant*)) OR (medicinal herb*)) OR (herbal product*)) OR (herbal plant*)) OR (herbal extract*)) OR (plant extract*)) OR (phytotherap*)) OR (botanical drug*)) OR (nutraceutical*)) OR (phytopharmaceutical*)) OR (natural product*)) OR (phytoconstituent*)) OR (phytochemical*)) AND (((((((hepatoprotect*) OR (liver protect*)) OR (liver disease*)) OR (cirrhosis)) OR (liver damage*)) OR (liver injur*)) OR (hepatotox*))) AND (((((((gut microbiota) OR (t microbiome)) OR (gut bacteria)) OR (gut flora)) OR (gut axis)) OR (gastrointestinal microbiota)) OR (gastrointestinal microbiome))	232

EMBASE

Set	Keywords	Results
**1**	'herbal medicine*':ab,ti OR 'medicinal plant*':ab,ti OR 'medicinal herb*':ab,ti OR 'herbal product*':ab,ti OR 'herbal plant*':ab,ti OR 'herbal extract*':ab,ti OR 'plant extract*':ab,ti OR 'phytotherap*':ab,ti OR 'botanical drug*':ab,ti OR 'nutraceutical*':ab,ti OR 'natural product*':ab,ti OR 'phytopharmaceutical*':ab,ti OR 'phytochemical*':ab,ti OR 'phytoconstituent*':ab,ti	210,384
**2**	'hepatoprotect*':ab,ti OR 'liver protect*':ab,ti OR 'liver disease*':ab,ti OR 'cirrhosis':ab,ti OR 'liver damage':ab,ti OR 'liver injury':ab,ti OR 'hepatotox*':ab,ti	400,119
**3**	'gut microbiota':ab,ti OR 'gut microbiome':ab,ti OR 'gut bacteria':ab,ti OR 'gut flora':ab,ti OR 'gut axis':ab,ti OR 'gastrointestinal microbiota':ab,ti OR 'gastrointestinal microbiome':ab,ti	65,303

**4**	('herbal medicine*':ab,ti OR 'medicinal plant*':ab,ti OR 'medicinal herb*':ab,ti OR 'herbal product*':ab,ti OR 'herbal plant*':ab,ti OR 'herbal extract*':ab,ti OR 'plant extract*':ab,ti OR 'phytotherap*':ab,ti OR 'botanical drug*':ab,ti OR 'nutraceutical*':ab,ti OR 'natural product*':ab,ti OR 'phytopharmaceutical*':ab,ti OR 'phytochemical*':ab,ti OR 'phytoconstituent*':ab,ti) AND ('hepatoprotect*':ab,ti OR 'liver protect*':ab,ti OR 'liver disease*':ab,ti OR 'cirrhosis':ab,ti OR 'liver damage':ab,ti OR 'liver injury':ab,ti OR 'hepatotox*':ab,ti) AND ('gut microbiota':ab,ti OR 'gut microbiome':ab,ti OR 'gut bacteria':ab,ti OR 'gut flora':ab,ti OR 'gut axis':ab,ti OR 'gastrointestinal microbiota':ab,ti OR 'gastrointestinal microbiome':ab,ti)	69

Web of Science

Set	Keywords	Results
**1**	((((((((((((TI=herbal medicine*) OR TI= (medicinal plant*)) OR TI=(medicinal herb*)) OR TI=(herbal product*)) OR TI=(herbal plant*)) OR TI=(herbal extract*)) OR TI=(plant extract*)) OR TI=(phytotherap*)) OR TI=(botanical drug*)) OR TI=(nutraceutical*)) OR TI=(phytopharmaceutical*)) OR TI=(natural product*)) OR TI=(phytoconstituent*)) OR TI=(phytochemical*)	85,940
**2**	((((((TI=hepatoprotect*) OR TI=(liver protect*)) OR TI=(liver disease*)) OR TI=(cirrhosis)) OR TI=(liver damage*)) OR TI=(liver injur*)) OR TI=(hepatotox*)	173,737
**3**	((((((TI=gut microbiota) OR TI=(gut microbiome)) OR TI=(gut bacteria)) OR TI=(gut flora)) OR TI=(gut axis)) OR TI=(gastrointestinal microbiota)) OR TI=(gastrointestinal microbiome)	35,853
**4**	((TS=((herbal medicine*) OR (medicinal plant*) OR (medicinal herb*) OR (herbal product*) OR (herbal plant*) OR (herbal extract*) OR (plant extract*) OR (phytotherap*) OR (botanical drug*) OR (nutraceutical*) OR (phytopharmaceutical*) OR (natural product*) OR (phytoconstituent*) OR (phytochemical*))) AND TS=((hepatoprotect*) OR (liver protect*) OR (liver disease*) OR (cirrhosis) OR (liver damage*) OR (liver injur*) OR (hepatotox*))) AND TS=((gut microbiota) OR (gut microbiome) OR (gut bacteria) OR (gut flora) OR (gut axis) OR (gastrointestinal microbiota) OR (gastrointestinal microbiome))	1464
